# Pan-American *Trypanosoma* (*Megatrypanum*) *trinaperronei* n. sp. in the white-tailed deer *Odocoileus virginianus* Zimmermann and its deer ked *Lipoptena mazamae* Rondani, 1878: morphological, developmental and phylogeographical characterisation

**DOI:** 10.1186/s13071-020-04169-0

**Published:** 2020-06-12

**Authors:** Herakles A. Garcia, Pilar A. Blanco, Adriana C. Rodrigues, Carla M. F. Rodrigues, Carmen S. A. Takata, Marta Campaner, Erney P. Camargo, Marta M. G. Teixeira

**Affiliations:** 1 Department of Parasitology, Institute of Biomedical Sciences, University of São Paulo, São Paulo, SP Brazil; 2 Department of Veterinary Pathology, Faculty of Veterinary Sciences, Central University of Venezuela, Maracay, Venezuela; 3 Fundación Esfera, Harpy Eagle Conservation Program in Venezuela, El Palmar, Bolívar Venezuela; 4 Earthmatters, Gainesville, FL USA; 5 Instituto Nacional de Ciência e Tecnologia, INCT-EpiAmo, Porto Velho, Rondônia Brazil

**Keywords:** *Trypanosoma*, New species, Cervidae, Deer ked, Phylogeny, Taxonomy, Great American Interchange, Host-parasite restriction

## Abstract

**Background:**

The subgenus *Megatrypanum* Hoare, 1964 of *Trypanosoma* Gruby, 1843 comprises trypanosomes of cervids and bovids from around the world. Here, the white-tailed deer *Odocoileus virginianus* (Zimmermann) and its ectoparasite, the deer ked *Lipoptena mazamae* Rondani, 1878 (hippoboscid fly), were surveyed for trypanosomes in Venezuela.

**Results:**

Haemoculturing unveiled 20% infected WTD, while 47% (7/15) of blood samples and 38% (11/29) of ked guts tested positive for the *Megatrypanum-*specific TthCATL-PCR. *CATL* and *SSU* rRNA sequences uncovered a single species of trypanosome. Phylogeny based on *SSU* rRNA and *gGAPDH* sequences tightly cluster WTD trypanosomes from Venezuela and the USA, which were strongly supported as geographical variants of the herein described *Trypanosoma* (*Megatrypanum*) *trinaperronei* n. sp. In our analyses, the new species was closest to *Trypanosoma* sp. D30 from fallow deer (Germany), both nested into TthII alongside other trypanosomes from cervids (North American elk and European fallow, red and sika deer), and bovids (cattle, antelopes and sheep). Insights into the life-cycle of *T. trinaperronei* n. sp. were obtained from early haemocultures of deer blood and co-culture with mammalian and insect cells showing flagellates resembling *Megatrypanum* trypanosomes previously reported in deer blood, and deer ked guts. For the first time, a trypanosome from a cervid was cultured and phylogenetically and morphologically (light and electron microscopy) characterised.

**Conclusions:**

In the analyses based on *SSU* rRNA, *gGAPDH*, *CATL* and ITS rDNA sequences, neither cervids nor bovids trypanosomes were monophyletic but intertwined within TthI and TthII major phylogenetic lineages. One host species can harbour more than one species/genotype of trypanosome, but each trypanosome species/genotype was found in a single host species or in phylogenetically closely related hosts. Molecular evidence that *L. mazamae* may transmit *T. trinaperronei* n. sp. suggests important evolutionary constraints making tight the tripartite *T. trinaperronei*-WTD-deer ked association. In a plausible evolutionary scenario, *T. trinaperronei* n. sp. entered South America with North American white-tailed deer at the Pliocene-Pleistocene boundary following the closure of the Panama Isthmus.
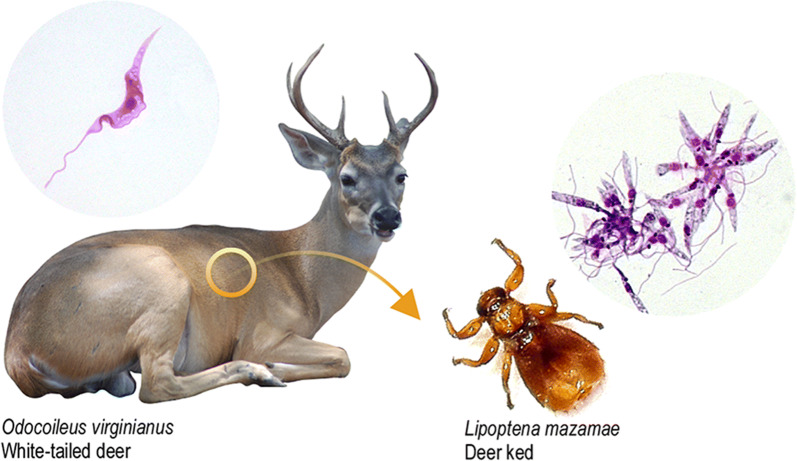

## Background

Trypanosomes of the subgenus *Megatrypanum* Hoare, 1964 of *Trypanosoma* Gruby, 1843 are distributed worldwide in bovids and cervids. Cervidae comprises 55 species widespread in Eurasia and America of two subfamilies: Cervinae, which comprises mostly deer from Eurasia of the genera *Axis*, *Dama*, *Elaphurus*, *Rucervus*, *Rusa* and *Cervu*s (the only genus also present in North America); and Capreolinae, which comprises deer from Eurasia (*Capreolus*), Eurasia and North America (*Alces*, *Rangifer*), and deer endemic to the Americas (*Odocoileus*) or restricted to South America (*Blastocerus*, *Hippocamelus*, *Mazama*, *Ozotoceros*, *Pudu*) [1].

In North American cervids, *Megatrypanum* trypanosomes have been reported in caribou (*Rangifer tarandus caribou* (Gmelin)) [2, 3], red deer (*Cervus elaphus* Linnaeus) [4], roe deer (*Capreolus capreolus* (Linnaeus)) [5–7], reindeer (*Rangifer tarandus* Linnaeus) [8], mule deer (*Odocoileus hemionus* (Rafinesque)) [3, 9], moose (*Alces alces* (Linnaeus)) [10], white-tailed deer (WTD) (*Odocoileus virginianus* (Zimmermann)) and elk (*Cervus elaphus canadensis* (Erxleben)) [3, 11, 12]. In Europe, *Megatrypanum* trypanosomes have been described in fallow deer (*Cervus dama* Linnaeus), red deer and roe deer in Germany [6], reindeer and moose in Sweden [13], roe deer in Poland [14], and red deer in Croatia [15]. In Asia, a *Megatrypanum* trypanosome was reported in the Japanese sika deer *Cervus nippon* Temminck [16]. *Megatrypanum* trypanosomes are generally non-pathogenic to domestic and wild ruminants including deer [8].

Contrasting with molecularly characterised *Megatrypanum* trypanosomes of deer from across the North America and Europe (Sweden, Germany, Croatia and Poland) [12–15], reports of these trypanosomes in South American cervids are limited to the morphology of blood trypomastigotes in WTD, black-tailed deer (*Odocoileus hemionus columbianus* (Richardson)) and brown brocket deer (*Mazama gouazoubira* (Fischer)) in Colombia [17, 18], and brocket deer in Argentina and Brazil [19, 20].

Traditionally, the large and broad shape of blood trypomastigotes is the main taxonomic criterion of the subgenus *Megatrypanum* (type-species *Trypanosoma* (*M.*) *theileri* Laveran, 1902 reported in domestic cattle), whereas species identification relies on data about host restriction provided by field, and cross-experimental infections [13, 21, 22]. This subgenus was revised by Hoare [21] to accommodate trypanosomes of artiodactyls, bats, rodents, non-human primates and marsupials, excluding trypanosomes of non-mammalian hosts formerly included in this subgenus. However, molecular phylogeny revalidated this subgenus as a monophyletic assemblage comprising the type-species *T. theileri* of cattle and virtually morphologically indistinguishable trypanosomes from bovids and cervids. Presently, this subgenus excludes all non-ruminant trypanosomes, even the poorly investigated closest relatives forming the *T. cyclops* sister clade [15, 23–25]. Although *T. theileri* was never observed in blood of hosts other than ruminants, PCR-surveys detected DNA of this trypanosome in bats [26], and in one chimpanzee [27].

Currently, there are three named species of *Megatrypanum* trypanosomes parasitizing deer, all based just on morphology and host species of origin: *Trypanosoma* (*M.*) *mazamarum* Mazza, Romana & Fiora, 1932 in brocket deer from Argentina [19]; *Trypanosoma* (*M.*) *cervi* Kingston & Morton, 1975 in elk from the USA [28]; and *Trypanosoma* (*M.*) *stefanskii* Kingston, Bobek, Perzanowski, Wita & Maki, 1992 in roe deer from Poland [14]. Molecular studies have uncovered different species/genotypes of *Megatrypanum* trypanosomes in WTD and elk in the USA [12], red deer in Croatia [15], sika deer in Japan [16], and the invader sika deer plus wisent (European bison), red deer and fallow deer in Poland [29].

*Megatrypanum* trypanosomes are thought to be cyclically transmitted by tabanid and hippoboscid flies. Trypanosomes similar to *T. cervi* have been reported in tabanids (deer flies) in the USA, Germany, Russia, and Poland [22, 29–31]. Tabanids harbouring trypanosomes of bovids have been reported in North America [32], South America [24, 33], and Africa [34–36]. The sheep ked *Melophagus ovinus* (Linnaeus, 1758) transmit *T.* (*M.*) *melophagium* Flu, 1908 exclusive of sheep [15, 21, 37] while *T.* (*M.*) *theodori* Hoare, 1931 of goats is transmitted by *Lipoptena capreoli* Rondani, 1878 [21]. The finding of trypanosomes in guts of *Lipoptena cervi* (Linnaeus, 1758) taken from red deer suggests that hippoboscid flies may transmit deer trypanosomes of the subgenus *Megatrypanum* [38]. However, molecular comparisons of *Megatrypanum* trypanosomes from deer blood and keds are still lacking.

Comprehensive molecular data on *Megatrypanum* trypanosomes from wild and domestic ruminants and the proper identification of vectors are essential to understand their evolution, species richness, phylogenetic relationships, range of vertebrate hosts, vectors, and geographical distribution. In the present study, we describe a new species of *Megatrypanum* in WTD and its deer ked and hypothesise its probable life-cycle and evolutionary history by integrating morphological, behaviour in cultures, biological and phylogeographical data.

## Methods

### Study area, deer blood and ked collection, and DNA preparation

**Fig. 1:**
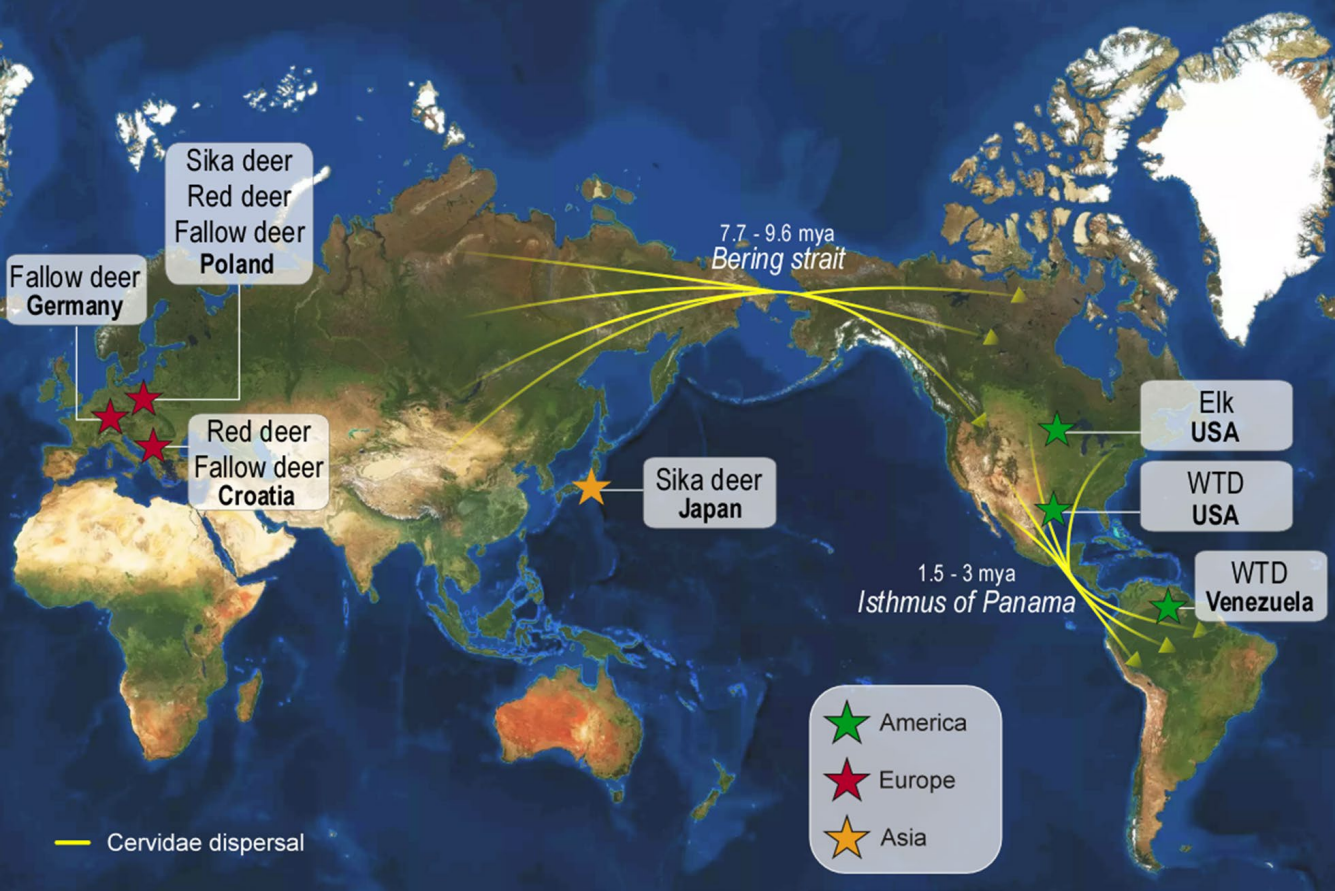
Origin of *Trypanosoma* (*Megatrypanum*) *trinaperronei* n. sp. and other deer trypanosomes. Geographical origin of deer examined in this and in previous studies, and historical dispersion of cervids from Eurasia reaching North America through the Bering Strait, and thereafter through the Panama Isthmus into South America. *Abbreviations*: WTD, white-tailed deer

**Fig. 2:**
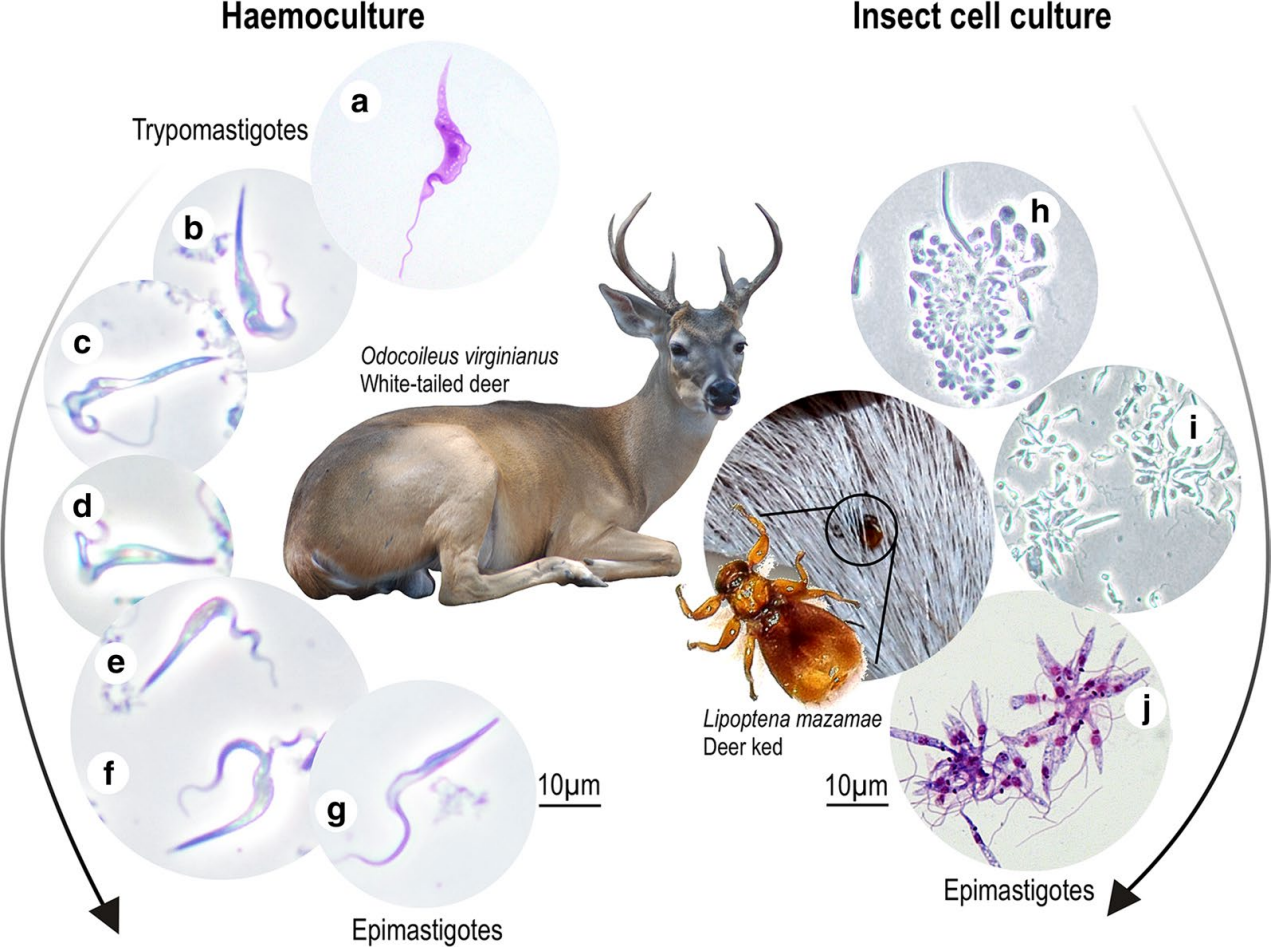
Predicted life-cycle of *Trypanosoma* (*Megatrypanum*) *trinaperronei* n. sp. in its host *Odocoileus virginianus* (white-tailed deer), and its putative vector the deer ked *Lipoptena mazamae* inferred from: early haemocultures showing long and slender trypomastigotes (**a**–**c**) and epimastigotes (**d**–**g**), both forms with noticeable undulant membrane; co-cultures with Hi-5 insect cells exhibiting clumps of small forms adhered to the insect cells (**h, i**) giving origin to rosettes of epimastigotes (**j**). Morphology and development of *T. trinaperronei* n. sp. co-cultivated with Hi-5 (25 °C) and LLCMK_2_ mammalian (37 °C), from log- to stationary cultures, are detailed in the Figs. 7 and 8. Deer keds become infected by *T. trinaperronei* n. sp. feeding on deer containing blood trypomastigotes resembling those present in early haemocultures (**a-c**), which, in their digestive tract, transform and multiply as small forms attached to the cells of the gut wall, as observed in Hi-5 cells (**h, i**), give origin to rosettes of epimastigotes (**j**) that multiply and, later, differentiate into metacyclic trypomastigotes. Illustration of *T. trinaperronei* n. sp. metacyclogenesis in insect cultures are shown in the Fig. 7. Most likely, infective metacyclic trypomastigotes present in the faeces of the vectors are transmitted to WTD by deer keds bite wound or mucosa, thus reaching the bloodstream and transforming into trypomastigotes resembling those detected in early haemocultures (**a**). *Abbreviations*: WTD, white-tailed deer

A total of 75 WTDs were captured at the Anzoátegui state, municipality of Simón Bolívar, an important livestock breeding area at north-eastern Venezuela (10°07′08.95″N, 64°38′23.80″W) (Fig. 1), where the mean annual temperature is 25 °C (21.8–2.2 °C) and rainfall is below 1000 mm, with a dry season from December to April. The captured WTDs (Fig. 2) were kept in quarantine prior to their introduction into a protected reserve. Chemical immobilization and anaesthesia were performed with a combination of 10 mg/kg ketamine and 0.6 mg/kg xylazine intramuscularly and reverted with 0.2 mg/kg of yohimbine intravenously. Anesthetized deer were examined to record heart and breathing frequency rates, and temperature. Institutional and national guidelines for the care and use of wild animals were followed. Deer handling was performed in accordance with the approved protocols, and under the supervision of the MINEC (the Venezuelan Ministerio del Poder Popular para el Ecosocialismo).

Blood samples were obtained *via* the jugular vein using tubes with EDTA and aliquots (~ 500 µl) of blood were preserved in 99.5% ethanol (v/v), incubated overnight in 250–500 μl of lysis buffer (20 mM EDTA; 50 mM Tris-HCL; 117 mM NaCl; 1% SDS; 10 mg/ml Proteinase K), precipitated with 400–800 μl ammonium acetate (4M), and centrifuged (10 min at 20,000× *g*). Then, DNA was precipitated with ethanol, dried at room temperature and resuspended in TE (Tris-EDTA). Deer keds (Fig. 2) were removed from the WTDs, preserved in ethanol, and identified based on morphology [39] and *cox*1 barcoding using DNA obtained from the gut contents of keds as described previously for tsetse flies [40]. Due to limited field facilities, the guts of deer keds were not examined by microscopy or culturing.

### Isolation of deer trypanosomes by hemoculturing

Deer blood samples were examined by microhematocrit, Giemsa-stained blood smears, and hemoculturing [15, 24, 25]. Aliquots (~ 200 µl) of blood were used for hemoculturing in medium consisting of blood agar base containing 15% rabbit blood as a solid phase and an overlay of TC100 medium (Cultilab, São Paulo, Brazil) with 10% FBS, and incubated at 25 °C [23, 24]. Positive haemocultures were transferred to culture flasks containing a monolayer of insect cells (Hi-5 from *Trichoplusia ni*) in TC100 medium with 10% FBS, and incubated at 25 °C [41]. Cultures were expanded in TC100 for DNA preparation and cryopreservation at the Trypanosomatid Culture Collection (TCC) of the Department of Parasitology, University of São Paulo (USP), São Paulo, Brazil.

### Trypanosome diagnosis in deer blood and keds and network of *CATL* sequences

The *Megatrypanum-*specific TthCATL-PCR based on Cathepsin-L (*CATL*) sequences was used for surveys in deer blood and ked guts as described previously [25, 42]. The cd*CATL* sequences (274 bp) of trypanosomes amplified by TthCATL-PCR were cloned and sequences determined were aligned with those of other *Megatrypanum* trypanosomes (GenBank, Additional file 1: Table S1), and used for network inferences [25, 42]. Sequences of the whole *CATL* catalytic domain (477 bp) were determined for the isolate TCC2268 [25, 42].

### Phylogenetic analysis of *SSU* rRNA, *gGAPDH* and ITS rDNA sequences

DNA from cultured trypanosomes extracted by the phenol-chloroform method was used for PCR amplification of sequences of the variable V7V8 region (~ 728 bp) or entire (2142 bp) *SSU* rRNA gene, *gGAPDH* (glycosomal glyceraldehyde 3-phosphate dehydrogenase) gene (~ 847 bp) and ITS1 rDNA sequences (~ 233 bp) [24, 25, 43]. PCR products were purified, cloned and sequences of 5–10 clones from each amplicon were determined, and aligned with sequences obtained from the GenBank using the Clustal X program [44]. We created the following alignments: (i) V7V8 *SSU* rRNA sequences herein determined and available on GenBank sequences for *Megatrypanum* trypanosomes; (ii) concatenated V7V8 *SSU* rRNA and *gGAPDH* sequences (~ 1575 bp) of *Megatrypanum* trypanosomes from deer, cattle, water buffalo, antelopes and sheep; trypanosomes of other subgenera were also included, and other trypanosomatids were used as outgroups; (iii) ITS1 rDNA sequences of *Megatrypanum* trypanosomes. Phylogenies were inferred using parsimony (P), maximum likelihood (ML), and Bayesian inferences (BI). P and bootstrap analyses were carried out using PAUP 4.0b10 [45] with 500 replicates of random addition sequences followed by branch swapping (RAS-TBR). ML was performed using RAxML-VI-HPC v.2.2.3 [46] with tree searches using GTR model with gamma-distributed rate variation across sites and proportion of invariable sites (GTRGAMMA model), and 500 maximum parsimony starting trees; the model parameters were estimated in RAxML for the duration of the tree search. Nodal supports were estimated with 500 bootstrap replicates in RAxML using GTRGAMMA and P starting trees. BI was performed in MrBayes v3.1.2 [47] with GTRGAMMA, and the first 25% of the trees from 1 million generations were discarded as ‛burn-inʼ. Sequences of ITS1 rDNA were employed for network split decomposition using the Neighbor-Net method with Kimura 2-parameter implemented in Splits Tree4 V4.10 [48]. Internode support was estimated with 500 bootstrap replicates using the same parameters optimized for network inferences.

Trypanosomes included in our analyses, and their host species, geographical origins and GenBank accession numbers of DNA sequences are detailed in Additional file 1: Table S1 (*CATL* sequences), Additional file 2: Table S2 (*SSU* rRNA gene), Additional file 3: Table S3 (*gGAPDH*) and Additional file 4: Table S4 (ITS1 rDNA). All newly generated DNA sequences were deposited in the GenBank database under the accessions numbers MN747149-MN747155 (*CATL*), MN752212 (*SSU* rRNA gene), MN756794 (*gGAPDH*) and MN752208-MN752209 (ITS1 rDNA).

### Growth behaviour and light and electron microscopy of trypanosomes co-cultivated with insect and mammalian cells

The isolate TCC2268 was co-cultivated with a monolayer of Hi-5 insect cells (TC100 medium) and flagellates from stationary cultures were seeded on monolayers of monkey LLC-MK_2_ cells, cultivated at 37 °C with 5% CO_2_, and intracellular parasites were investigated according to Lima et al. [49]. Both cultures were examined daily in an inverted microscopy, and supernatants of 2, 5, 7 and 10 days of culture were smeared on glass slides, fixed with methanol, and stained with Giemsa for light microscopy.

Both scanning electron microscopy (SEM) and transmission electron microscopy (TEM) were performed as detailed in [41]. For SEM, trypanosomes fixed in glutaraldehyde were adhered to poly-L-lysine-coated coverslips and processed for observation on a FEI Quanta 250 (FEI Company, Hillsboro, USA) microscope. For TEM, trypanosomes were fixed in glutaraldehyde, post-fixed with osmium tetroxide, and embedded in Spurr resin. Ultrathin sections were stained with uranyl acetate and lead citrate, and examined with a LEO 906E microscope (Zeiss, Jena, Germany). Images were captured by a CCD camera MegaView III.

## Results and discussion

### White-tailed deer and deer keds harbour *Megatrypanum* trypanosomes uncovered by TthCATL-PCR

Overall, 15 WTDs including 9 males (5 juveniles and 4 adults) and 6 juvenile females captured were clinically healthy, showing normal values of heart frequency (44 to 128, mean of 76, beats/min), breathing frequency (18 to 48, mean of 34, breaths/min), and body temperature (35.4–40.2 °C, mean of 37.4 °C). Inspection of WTDs for ectoparasites revealed an abundance of deer keds in ~ 73% (11/15) of them, mainly in the ventral, inner legs and inguinal regions of the WTDs. No skin or fur damage was observed in ked-infested WTDs. Twenty-nine keds from 11 WTDs preserved in ethanol were identified morphologically as *L. mazamae* [39], and this identification was confirmed by *cox*1 DNA barcoding (GenBank: MN756795).

Microscopy of Giemsa-stained blood smears and the microhematocrit technique did not allow detection of trypanosomes in blood samples of the 15 WTDs examined. However, haemoculturing yielded a trypanosome infection rate of 20% (three positive cultures), and one culture (TCC2268) was established, and cryopreserved. These findings are consistent with very low parasitaemia but positive haemocultures as previously reported for other *Megatrypanum* trypanosomes [23, 25]. The *Megatrypanum-*specific assay, TthCATL-PCR, was employed aiming at detection of trypanosomes in the blood samples from WTD and in gut contents from the deer keds. PCRs were positive for 7 out of 15 WTDs (~ 47%), and 11 out of 29 deer keds (~ 38%), including keds from the three WTD positive for trypanosomes by haemoculturing.

### Characterisation of trypanosomes from blood and keds of white-tailed deer using *CATL* sequences

*CATL* DNA sequences (274 bp) obtained by TthCATL-PCR from blood samples and culture (isolate TCC2268) of WTD and gut samples from deer keds share highest similarity with sequences of *Megatrypanum* trypanosomes on GenBank. The newly generated sequences were aligned with those of other *Megatrypanum* trypanosomes, and this alignment was used to infer their genetic relatedness. Trypanosome sequences obtained from deer keds were virtually identical to those obtained from WTD blood (TCC2268), indicating that they belong to a single trypanosome species.

**Fig. 3:**
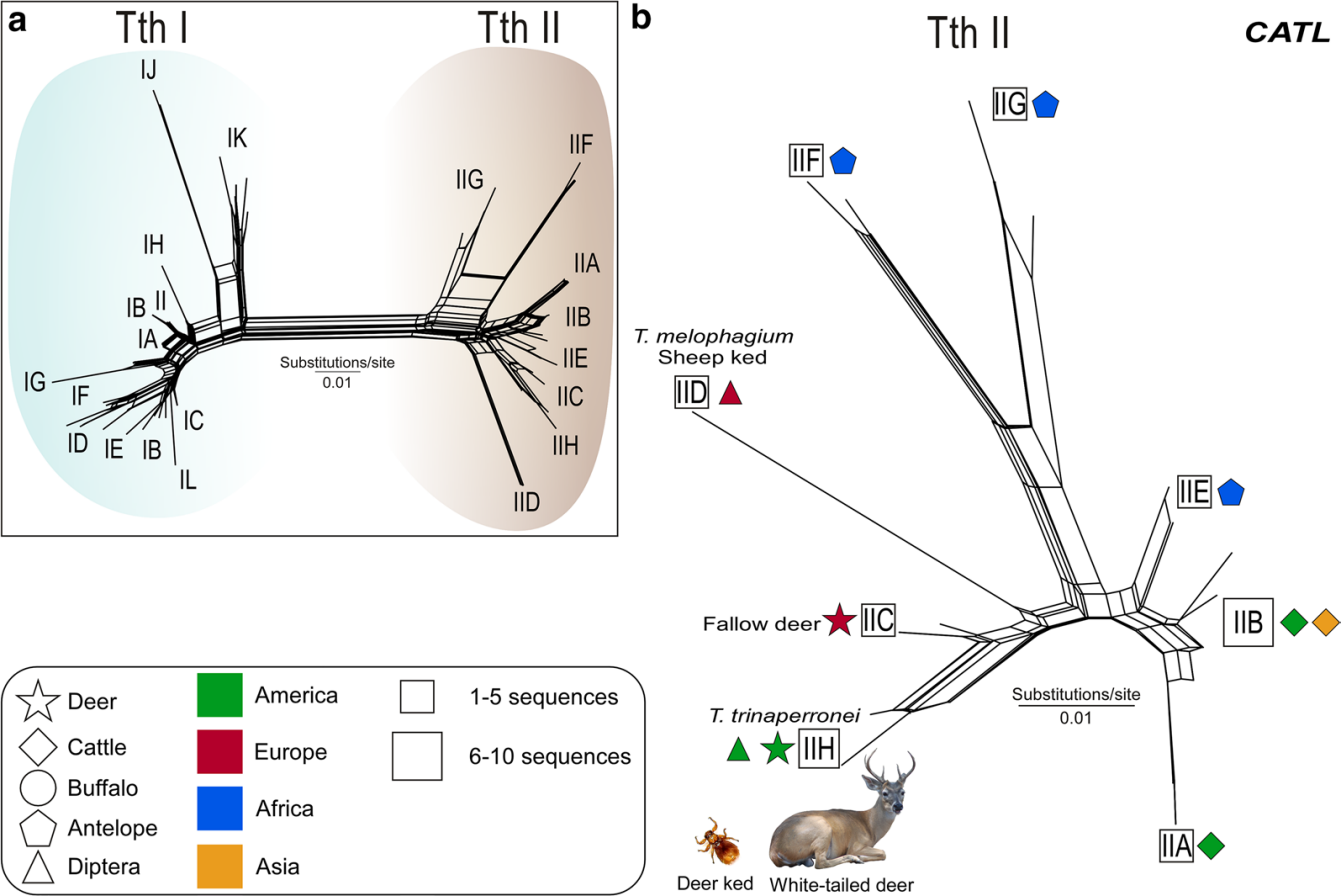
Network analysis of cathepsin L (*CATL*) sequences of *Trypanosoma* (*Megatrypanum*) *trinaperronei* n. sp. from Venezuelan white-tailed deer (WTD), and respective deer keds. **a** Analysis of *Megatrypanum* trypanosomes from wild and domestic ruminants evidencing TthI and TthII phylogenetic lineages. **b** Analysis restricted to the lineage TthII positioning *T. trinaperronei* n. sp. (TCC2268) tightly clustered with trypanosomes found in guts of deer ked taken from WTD. Analysis was inferred using the Neighbour-Net method with the K2 parameter and nodal support estimated with 500 bootstrap replicates. *Abbreviations*: TCC, Trypanosomatids Culture Collection; WTD, white-tailed deer

Network inferences of 100 *CATL* sequences of 274 bp, amplified by TthCATL-PCR (Fig. 3a, b) or 53 larger *CATL* sequences of 477 bp (data not shown) generated highly similar networks. A divergence of ~ 2.0% in the small fragment of *CATL* sequences separated TCC2268 from *Trypanosoma* sp. D30. Although originating from different continents, these two deer isolates were much more related to each other than to trypanosomes from bovids (cattle, buffalo, sheep and antelopes), even those from Venezuelan cattle and buffalo [25]. TCC2268 and *Trypanosoma* sp. D30 were assigned to different species of close genotypes, TthII H (which includes *CATL* sequence from deer ked) and TthII C, respectively. TCC2268 diverged by 5.3% from *T. melophagium* (TthII D) and by 3.3% from *T. theileri* of cattle nested into TthII (Fig. 3b).

Although useful for assessment of genetic diversity [42, 50–52], the low level of polymorphism detected in short *CATL* sequences (274 bp) generated by TthCATL-PCR may be insufficient to discriminate reliable lineages and, specially, genotypes. Therefore, the description of novel species and intraspecific genotypes must be supported by additional markers including conserved (*SSU* rRNA and *gGAPDH*), and polymorphic (ITS rDNA and spliced leader rRNA) sequences [15, 25, 42, 43, 50, 53].

### Barcoding of deer trypanosomes through *SSU* rRNA sequences revealed a new *Megatrypanum* trypanosome in white-tailed deer

**Fig. 4:**
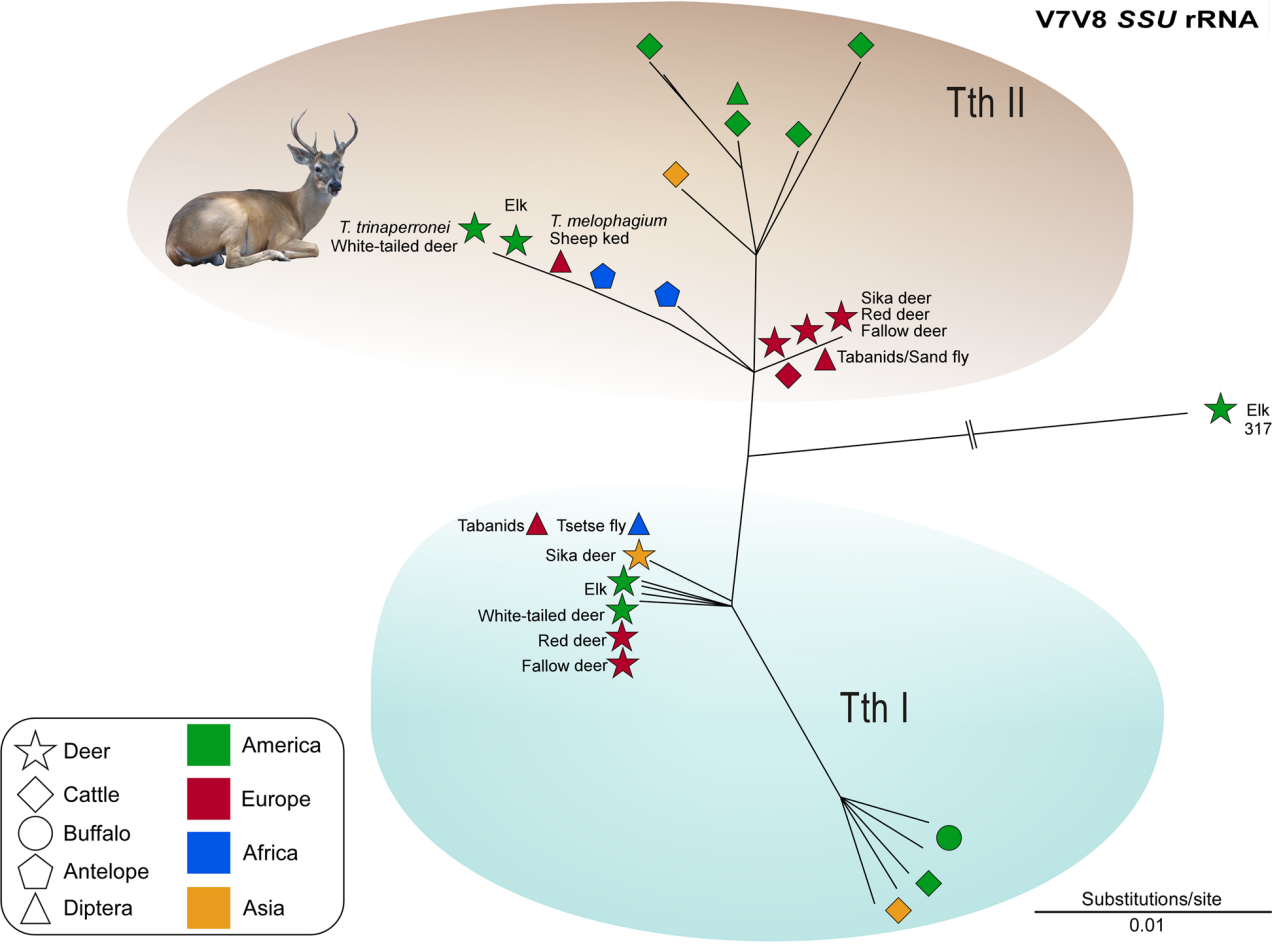
Barcoding of *Trypanosoma* (*Megatrypanum*) *trinaperronei* n. sp. Comparison of V7V8 *SSU* rRNA barcodes from *T. trinaperronei* n. sp. and trypanosomes of TthI and TthII lineages of the subgenus *Megatrypanum* from cervids (fallow, red and sika deer, WTD, and elk), and bovids (water buffalo, cattle, bison, and antelopes). Sequences obtained from deer keds, sheep keds, sand flies, tsetse flies and tabanids were also included. Sequences from a distantly related isolate of elk (elk 317) from the USA were placed as a probable new lineage of *Megatrypanum*. Dendrograms were inferred by Neighbour-Joining using the Kimura 2-parameter algorithm, and nodal supports were estimated with 500 bootstrap replicates. *Abbreviations*: WTD, white-tailed deer

Our comparative analysis of V7V8 *SSU* rRNA barcodes comprised *Megatrypanum* trypanosomes from cervids (Venezuela, Germany, Poland, Croatia, Japan and USA), and a large dataset of trypanosome sequences from bovids including cattle (Brazil, Venezuela, Argentina, Colombia, Germany, Poland, Croatia, UK, Japan and USA), water buffaloes (Venezuela, Colombia and Brazil), antelopes (Cameroon and Tanzania) and bison (Poland). In addition, our analyses included sequences of *Megatrypanum* trypanosomes from the guts of tabanids (Brazil, Africa and Russia), hippoboscids (Croatia and Scotland), tsetse flies (Africa), and sand flies (Italy) (Fig. 4; Additional file 2: Table S2). *SSU* rRNA barcodes corroborated the distribution of cervid and bovid trypanosomes in both TthI and TthII lineages, whereas the branching patterns of intra-lineages could not be resolved using exclusively these highly conserved sequences (Fig. 4). In addition, two sequences from an elk (elk 317; GenBank: JX178200, JX178201) from the USA diverged by relevant and equidistant genetic distances from TthI (~ 3.0% divergence) and TthII (~ 3.1%), apparently representing a new lineage of *Megatrypanum* (Fig. 4).

*SSU* rRNA sequences of the Venezuelan isolate WTD TCC2268 was highly similar (99.7%) to that of the isolate WTD A3 from the USA, both sharing 99.5% similarity with *Trypanosoma* sp. D30 of fallow deer from Germany. Besides these three deer trypanosomes, TthII comprised *Trypanosoma* cf*. cervi* from North American elk (elk328), and isolates from red deer (Cel34), fallow deer (DdP18) and sika deer (Cn1) from Poland. In addition, TthII included trypanosomes from African antelopes and *T. melophagium* of sheep, all clustering close to the trypanosomes of deer, whereas trypanosomes of cattle from North and South America, Europe and Asia formed a more separated cluster (Fig. 4). Trypanosomes from these European deer nested into TthII diverged from TCC2268 by 0.3–0.6% in highly conserved *SSU* rRNA sequences. For comparison, TCC2268 diverged by just 0.6% from the reference sequence for *T. theileri* TREU124 of cattle, thus reinforcing that these sequences are too conserved to clearly resolve the relationships of the recently diversified *Megatrypanum* trypanosomes. Regarding possible vectors, trypanosome sequences obtained from the guts of Brazilian, Polish and Russian tabanids, and Central African Republic tsetse flies were all assigned to both TthI and TthII lineages [24, 29, 36].

The lineage TthI also harboured trypanosomes (misclassified) referred to as *Trypanosoma* cf*. cervi* identified in WTDs (WTD A1, A5, A21, A148 and NL15) and elk (elk142, elk328, elk416 and elk421) from the USA [12], and isolates referred to as *T. cervi* from Polish red deer (Cel14St), fallow deer (DdP287) and tabanids (Fig. 4). The trypanosome (TSD1) from the Japanese sika deer [16] was the deer trypanosome more genetically distant (~ 1.4% sequence divergence) from the isolate TCC2268 (Fig. 4). In addition, this lineage also comprised *T. theileri* of cattle from South America, Japan and the USA, and *T. theileri-*like trypanosomes of South American water buffalo and European bison from Poland [24, 25, 29].

### Phylogeny based on *SSU* rRNA and *gGAPDH* genes supports a new species of trypanosome from white-tailed deer in the subgenus *Megatrypanum*

It was previously demonstrated that *gGAPDH* sequences are generally more variable than *SSU* rRNA sequences, thus being more suitable for a better differentiation between closely related trypanosomes such as those of the subgenus *Megatrypanum* [15, 24, 25]. Corroborating results using *SSU* rRNA sequences, the *gGAPDH* sequence from the isolate WTD TCC2268 was closest to *Trypanosoma* sp. D30 (~ 2.2% sequence divergence), and more similar to *T. melophagium* and trypanosomes of antelopes, all clustering together in the lineage TthII. Cattle isolates formed another group within TthII, separated from WTD TCC2268 by an average of ~ 4.4% sequence divergence.

**Fig. 5:**
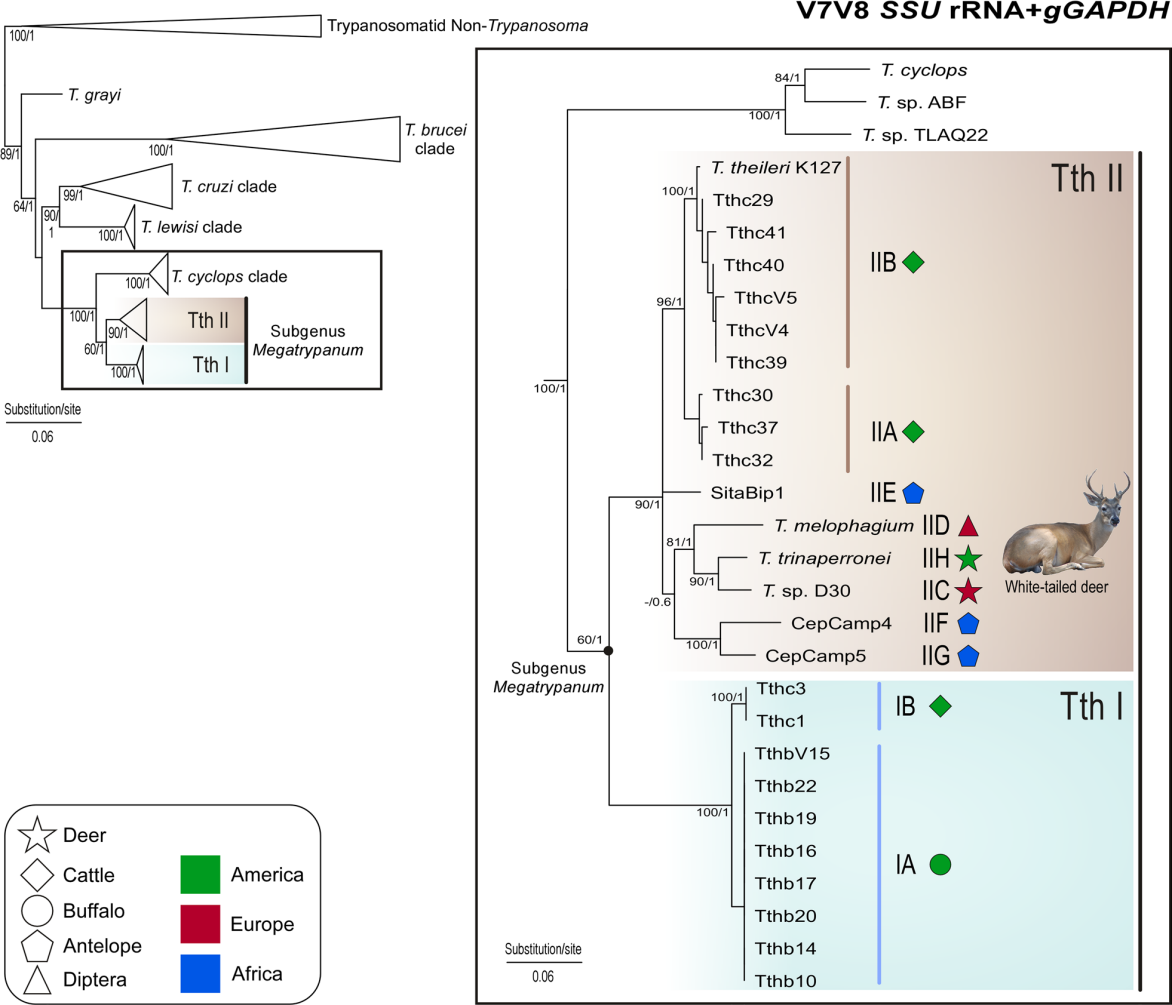
Phylogenetic positioning of *Trypanosoma* (*Megatrypanum*) *trinaperronei* n. sp. **a** Phylogenetic tree based on concatenated *gGAPDH* and *SSU* rRNA sequences from *Megatrypanum* trypanosomes and species representative of all other major clades of *Trypanosoma*, using trypanosomatids of other genera as outgroups. Major phylogenetic clades were collapsed, and the clade *Megatrypanum* was highlighted. **b***Megatrypanum* clade showing TthI and TthII lineages formed by several genotypes. *Trypanosoma trinaperronei* n. sp. was assigned to genotype TthII H, sister to TthII C, which comprised the German *Trypanosoma* sp. D30 of fallow deer and the Croatian TC2 of red deer, altogether forming a clade exclusive of deer trypanosomes from South and North America, and Europe. A concatenated alignment of 1575 characters was employed for maximum likelihood (ML) and Bayesian inference (BI); the numbers at nodes refer to ML/B support values derived from 500 replicates

Here, *gGAPDH* sequences of TCC2268 were aligned with available sequences from species/genotypes of *Megatrypanum* trypanosomes, and concatenated with *SSU* rRNA sequences. The inferred phylogeny (Fig. 5) displayed a highly congruent topology compared with that of independent *SSU* rRNA (Fig. 4). Together, phylogenetic positioning and genetic distances of WTD TCC2268 from other species of *Megatrypanum* with sequences available on GenBank allowed for the description of this new isolate as a novel species herein designated *Trypanosoma* (*Megatrypanum*) *trinaperronei* n. sp. Unfortunately, most trypanosomes from cervids have been known merely by partial *SSU* rRNA sequences such as those reported from elk and WTD from the USA, and deer isolates from Japan and Poland included in the present study in the *SSU* rRNA analysis (Fig. 4). Results obtained in our analyses are congruent with those from previous studies using *SSU* rRNA, *gGAPDH*, *CATL* and ITS rDNA sequences [15, 24, 25, 42, 50, 53].

### Low levels of polymorphism in ITS1 rDNA sequences of *T. trinaperronei* n. sp. of white-tailed deer from Venezuela and the USA

**Fig. 6:**
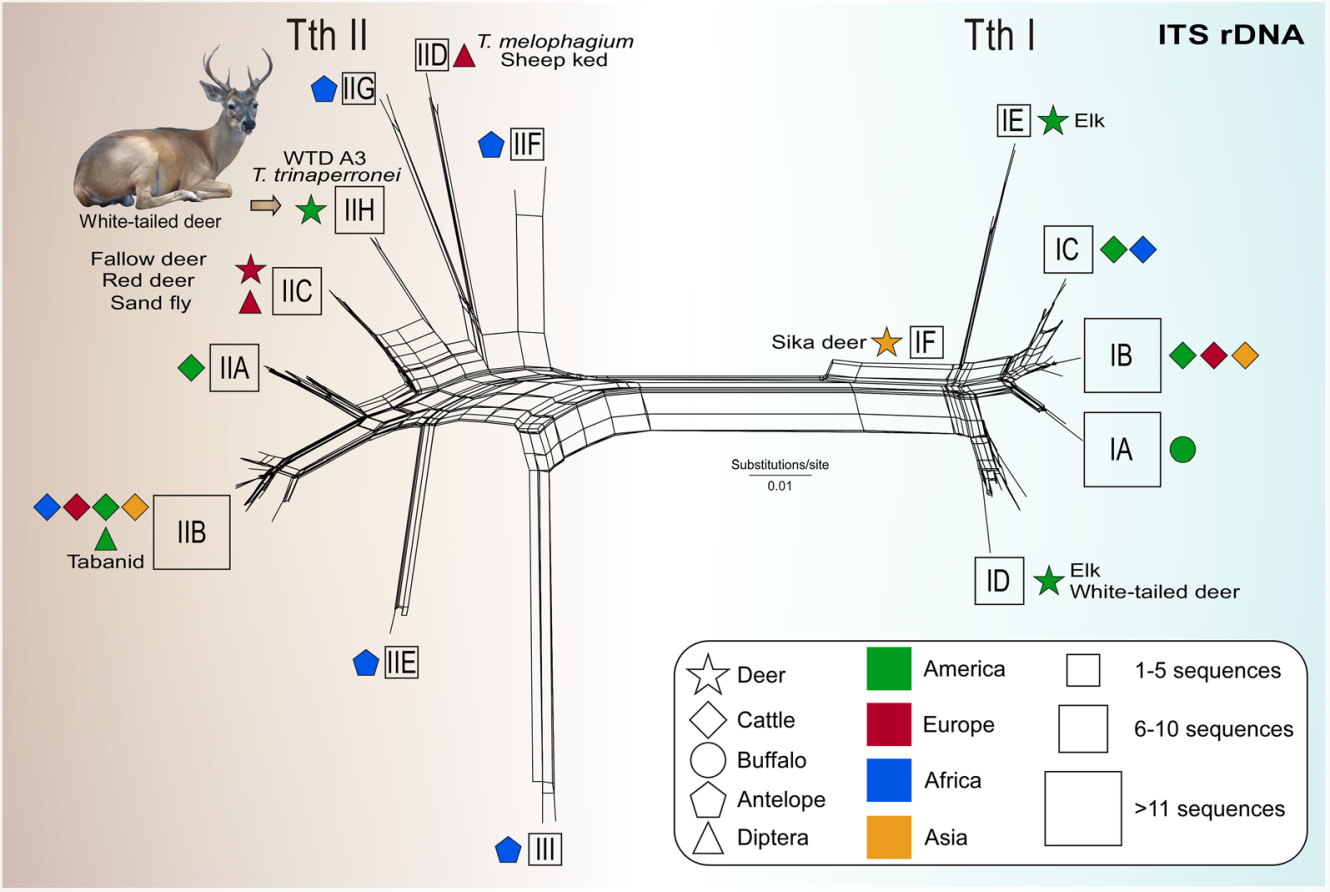
Lineages and genotypes of trypanosomes of the subgenus *Megatrypanum* inferred using ITS1 rDNA sequences. Network inferred using polymorphic ITS1 rDNA sequences of trypanosomes representative of genotypes identified in cervids (WTD, follow deer, sika deer and elk), bovids (cattle, water buffalo and antelopes), sheep keds, and sand flies. The analysis was carried out using the Neighbour-Net method with K2P parameter, and nodal support estimated with 500 bootstrap replicates. *Trypanosoma trinaperronei* n. sp. comprised two highly similar isolates, TCC2268 from Venezuela and WTD A3 from Texas (USA) assigned to the new genotype TthII H, which is closest to TthII C formed by *Trypanosoma* sp. D30 of fallow deer and TC2 of red deer from German and Croatia, respectively. *Abbreviations*: TCC, trypanosomatids culture collection; WTD, white-tailed deer

To better understand the relatedness of *T. trinaperronei* n. sp. with its closely related trypanosomes of deer from the USA and Germany, we compared ITS1 rDNA sequences, which are much more polymorphic than *SSU* rRNA and *gGAPDH* sequences. A comprehensive analysis of 122 ITS1 rDNA sequences of *Megatrypanum* trypanosomes (Fig. 6) was carried out including sequences of trypanosomes from a range of deer species and geographical areas: WTD TCC2268 from Venezuela (*n* = 2 sequences); WTD A3 (*n* = 2), WTD A1 (*n* = 2), WTD A21 (*n* = 3), WTD NL15 (*n* = 3), elk 416 (*n* = 1), elk 142 (*n* = 2), elk 421 (*n* = 1), and elk 328 (*n* = 1) from the USA; TSD1 (*n* = 1) from Japanese sika deer; TC2 (*n* = 1) from Croatian red deer; and *Trypanosoma* sp. D30 from German fallow deer (*n* = 2). These sequences were aligned with those from bovid trypanosomes: *T. theileri* of cattle (29 sequences of TthI and 37 of TthII lineage); *T. theileri-*like of water buffalo (*n* = 13); *T. melophagium* of sheep ked; and trypanosomes of the antelopes (*n* = 14) sitatunga, duiker and puku.

Confirming previous studies, ITS1 rDNA sequences of *Megatrypanum* trypanosomes were distributed in the TthI and TthII lineages (Fig. 6) regardless of their origin from bovids or cervids, thus corroborating data from the present study (Figs. 3, 4 and 5) and previous studies [12, 15, 24, 25, 53]. Our analysis supported 6 genotypes of TthI (IA-IF), and 9 of TthII (IIA-II I) (Fig. 6). ITS1 rDNA sequences of *T. trinaperronei* n. sp. from Venezuela and the USA shared highly similar sequences (~ 0.6% divergence), and were tightly clustered together supporting the genotype TthII H. Trypanosomes from European cervids assigned to the lineage TthII C were the German *Trypanosoma* sp. D30 of fallow deer [12, 25] and the highly similar Croatian trypanosome of red deer [15], and they diverged ~ 17% from *T. trinaperronei* n. sp. Regarding the relationships between cervid and bovid trypanosomes, ITS1 rDNA sequences of *T. melophagium* diverged by ~ 18% from *T. trinaperronei* n. sp. while divergences of above 24% separated *T. trinaperronei* n. sp. from trypanosomes of cattle (TthII A and TthII B) and antelopes (TthII F, G, E, I) [25]. Interestingly, ITS1 rDNA sequence of a trypanosome obtained from the gut of an Italian sand fly [54] was virtually identical to those of *Trypanosoma* sp. D30 from Germany [22]. As expected, deer trypanosomes of TthI diverged from *T. trinaperronei* n. sp. by remarkable divergences in ITS rDNA (Fig. 6): ~ 46% from both TthI D (WTD and elk) and TthI E (elk), which are genotypes identified in deer sympatric with the WTD from which *T. trinaperronei* n. sp. (isolate WTD A3) was obtained in the USA [12]. As shown with other molecular markers here, the greatest distances of ITS rDNA (~49%) among deer trypanosomes were observed between *T. trinaperronei* n. sp. and the trypanosome from Japanese sika deer (TthI F).

### Host-parasite-vector relationships and evolutionary history of cervid trypanosomes

Although host specificity of *Megatrypanum* trypanosomes remains to be clearly demonstrated, our findings provide additional support for relevant host-parasite-vector association in the evolution of these trypanosomes. Species diversification was likely shaped by evolutionary constraints exerted by ruminant hosts. In addition, vectors may be also involved in trypanosome host-restriction because deer flies (tabanids) and deer keds (hippoboscids) are strongly associated with their cervid hosts. In agreement with this hypothetical evolutionary scenario, we demonstrated that deer keds taken from WTD exclusively harboured *T. trinaperronei* n. sp., corroborating a previous suggestion that these flies can transmit, cyclically and/or mechanically, *Megatrypanum* trypanosomes to cervids [38]. *Lipoptena mazamae* occurs from south-eastern USA to South America [39, 55] and is tightly linked to WTD, although this ked can eventually jump to phylogenetically close deer species [39]. To date, deer flies, which have been experimentally proven to transmit deer trypanosomes, and deer keds have been implicated as vectors of cervid trypanosomes [21, 29, 31, 36, 38, 56, 57]. Recent studies report on DNA from deer trypanosomes in guts of sand flies and culicids [54, 58], but their roles as vectors remains to be investigated.

Trypanosome cross-infections of cervids and bovids have not been confirmed experimentally or by molecular epidemiology [8, 12, 15, 21, 24, 25, 33, 53, 59]. In Venezuela, we found WTD infected with *T. trinaperronei* n. sp. while sympatric cattle and water buffalo were found infected with *T. theileri* and *T. theileri-*like, respectively [25]. Similarly, Japanese sika deer were found infected exclusively with the *Trypanosoma* sp. TSD1, whereas sympatric cattle were infected with *T. theileri* of both TthI and TthII lineages [53]. Deer, cattle and sheep have been reported to harbour host-specific trypanosomes in Croatia [15]. All these findings, coupled with data herein reported, provide strong evidence that *Megatrypanum* trypanosomes exhibit a narrow host range or even host specificity. Each trypanosome species/genotype was found in a single host species or in closely phylogenetically related hosts, those found in cervids were never detected in bovids, although one host species can harbour trypanosomes of more than one species or genotype [12, 23–25, 53]. To date, reports of elk and WTD sharing trypanosome genotype [12] relied merely on DNA detection, and genuine infections remain to be demonstrated. Reports based on exclusively on morphology of *T. cervi*, originally in an elk [28], and subsequently in a range of deer including WTD, wapitis [28], mule deer [9], moose [10] and reindeer in the USA [8], and in European fallow, roe and red deer [6] must be all molecularly confirmed.

It has been demonstrated by isoenzyme and karyotype analyses that the trypanosome found in Swedish reindeer differ from those found in moose, and both differed from cattle isolates, despite all these animals living in sympatry [13]. Similarly, data from zymodemes suggested the existence of different species of *Megatrypanum* infecting distinct species of deer and cattle in Germany [59]. The isolates of *T. trinaperronei* n. sp. from Venezuela and the USA are closely related, but not identical. Interestingly, *T. trinaperronei* n. sp. is more related to deer trypanosomes from Germany, Croatia, Poland and Russia, all nested into TthII lineage [15, 29, 31, 54], than to trypanosomes found in sympatric WTD and elk (USA) nested into TthI, a lineage also harbouring a trypanosome of Japanese sika deer [12, 16].

Our findings agreed with multiple and relatively recent crossings of the Bering Strait by cervids infected with *Megatrypanum* trypanosomes reaching North America from Eurasia, and from these regions dispersing through the world. Altogether, deer-trypanosome-vector associations and phylogeography support a plausible evolutionary scenario where WTD infected with the ancestor of *T. trinaperronei* n. sp., likely infested by its tightly linked ectoparasite *L. mazamae*, were introduced from North America into South America through the Panama Isthmus, reaching this continent at the Pliocene-Pleistocene boundary [1, 60]. Cervidae originated in Asia between 7.7 and 9.6 mya, and according to fossil records, deer did not cross the Bering Land Bridge to North America before 4.2 to 5.7 mya [60]. South American cervids are thought to have originated from at least two invasion events by North American deer: first, by the common ancestor of all deer species endemic to South America during the Great American Interchange at the Early Pliocene (~ 3 mya), and more recently (~ 1.5 mya) by WTD at the Pliocene-Pleistocene boundary [1, 60]. Unfortunately, the only trypanosome reported in a deer species endemic to South America is *T. mazamarum*, described in the blood of the brocket deer in Argentina [19], and never cultivated or molecularly characterized.

Concordant with our data on *Megatrypanum* trypanosomes, host-helminth assemblages were also associated with an early dispersion of cervids and bovids from Eurasia into North America, and then into the Neotropics [61]. Also supporting the recent dispersion of cervids and their parasites, *Plasmodium* sp. from the South American pampas deer (*Ozotoceros bezoarticus*) is closely related to *Plasmodium odocoilei* of North American WTD, and these two species are estimated to have diverged just by 0.3–0.9 mya [62].

### Development of *T. trinaperronei* n. sp. in haemocultures and cultures with insect and mammalian cells

In early (7–10 days) haemocultures of WTD blood, live flagellates (phase-contrast microscopy) exhibited a few trypomastigotes (Fig. 2a–c) with large body length, pointed posterior ends and noticeable undulating membrane, alongside large transition forms between trypomastigote and epimastigote forms (Fig. 2d, e, g), and dividing epimastigotes (Fig. 2f). Flagellates of *T. trinaperronei* n. sp. from early haemocultures seeded on monolayers of insect cells (Hi-5), at 25 °C, initially formed clumps of small and rounded forms attached to insect cell membranes (Fig. 2h, i); these forms increased in length to became epimastigotes that remained adhered by their flagella forming rosettes until released into the supernatant of cultures (Fig. 2j). The developmental forms of *T. trinaperronei* n. sp. co-cultivated with Hi-5 insect cells very much resembled those reported for *T.* (*Megatrypanum*) spp. in the gut of the ked *L. cervi* taken from red deer [38] and *T. melophagium* adhered to the cells of gut walls of sheep keds [15, 21].

**Fig. 7:**
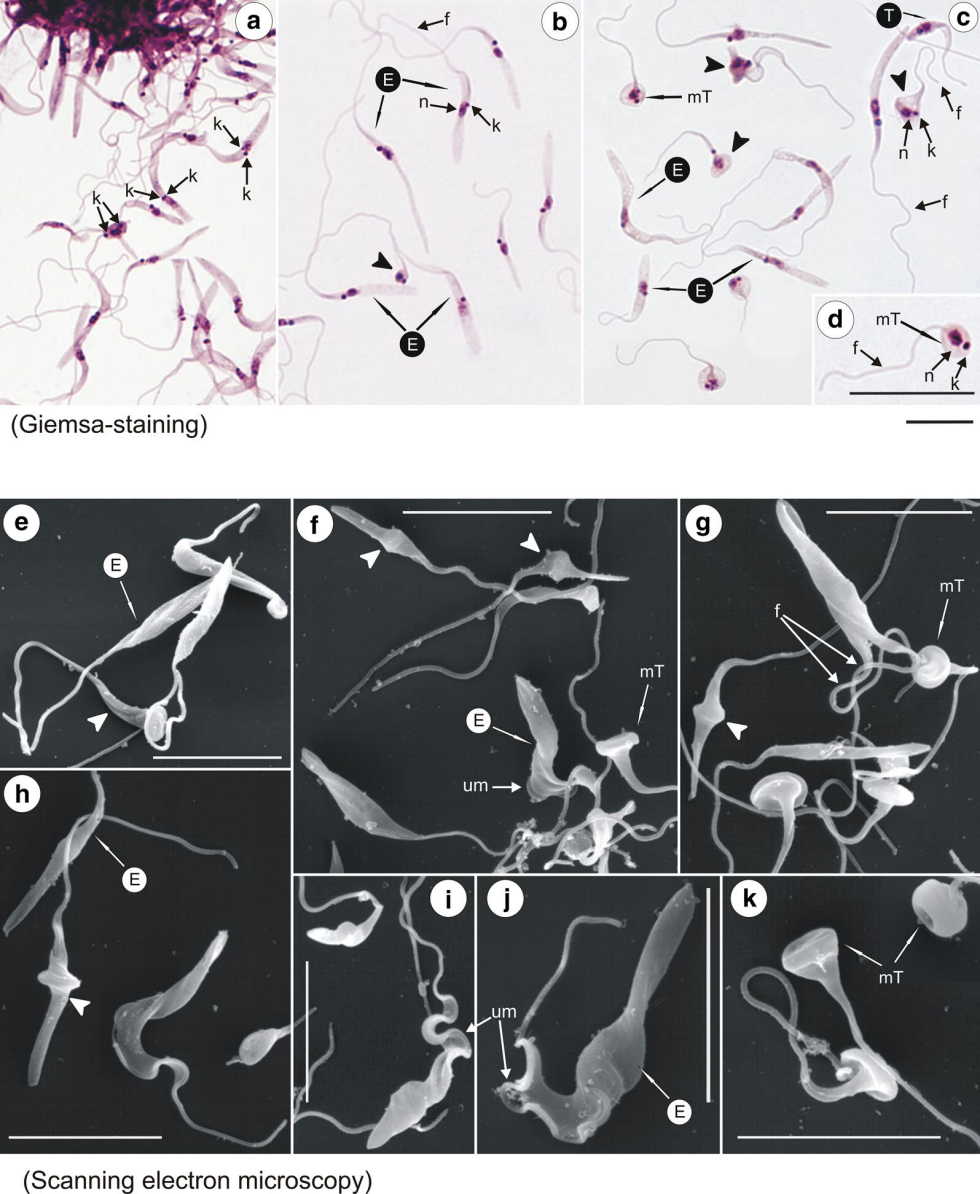
Light and scanning electron microscopy (SEM) of *Trypanosoma trinaperronei* n. sp. co-cultured with insect cells at 25 °C. Giemsa-stained (**a**–**c**) forms of a log-phase (5 days) culture (**a**) exhibiting flagellates adhered by their flagella forming rosettes, and detached epimastigotes. **b** Mid-log (7 days) cultures showing long and thin epimastigotes. **c** Stationary culture (10 days) exhibiting epimastigotes, transition forms between epi- and trypomastigotes (arrow heads), and unique bell-shaped metacyclic trypomastigotes. A typical metacyclic was enlarged in (**d**). SEM of mid-log cultures (7 days) showing epimastigotes of variable length and width (**e**–**j**) including forms with well-developed undulant membranes (**f**, **j**), transition forms (**f**–**h**), and bell-shaped metacyclic trypomastigotes (**f**, **g**, **k**). *Abbreviations*: nucleus, n; kinetoplast, k; flagellum, f; undulating membrane, um; epimastigote, E; trypomastigote, T; metacyclic trypomastigotes, mT. *Scale-bars*: **a**–**k**, 10 µm

Epimastigotes of *T. trinaperronei* n. sp. in log phase Hi-5 cultures (5 days) multiplied intensively attached by their flagella forming large rosettes (Fig. 7a), which initially remained adhered to the insect cells and afterwards were released in the supernatant, where free epimastigotes became progressively abundant (Fig. 7a, b). Giemsa-stained epimastigotes showed the rounded kinetoplast adjacent and lateral to the central nucleus with an almost imperceptible undulating membrane, and a long free flagellum (Fig. 7a, b). In mid-log cultures (7 days), most epimastigotes became longer and thinner with a pointed posterior extremity (Fig. 7b). Stationary phase cultures (10 days) of *T. trinaperronei* n. sp. exhibited variable forms, all with a long free flagellum, including some wider epimastigotes exhibiting more preeminent undulating membranes (Fig. 7c). Some forms became progressively shortened in their posterior ends giving origin to blunted forms (indicated by arrowheads in Fig. 7c) during the differentiation of epimastigotes to trypomastigotes (Fig. 7b, c) and, finally, to ‛roundedʼ forms with a long flagellum (Fig. 7b, c), which most likely represented metacyclic trypomastigote forms (Fig. 7d). In contrast with the slow movement of the long epimastigotes, these ‛roundedʼ forms were highly mobile, and resembled metacyclic trypomastigotes of *T. theileri* described previously in the guts of tabanid flies and stationary cultures [57]. Overall, initial co-cultivation of *T. trinaperronei* n. sp. with insect cells, at 25 °C, showed flagellates resembling those of *Trypanosoma* (*Megatrypanum*) spp. present in the guts of *L. cervi*, the Old-World deer ked, collected from red deer [38].

Scanning electron microscopy (SEM) of the mid-log cultures (7 days) of *T. trinaperronei* n. sp. in Hi-5 cultures showed flagellates of variable length and shape (Fig. 7e–j): slender epimastigotes without a noticeable undulating membrane (Fig. 7e) became broader epimastigotes exhibiting a conspicuous undulating membrane, easily detectable by SEM (Fig. 7f, i, j). Following the differentiation from epimastigotes to trypomastigotes, a range of transition forms (indicated by arrowheads in Fig. 7f–h) were observed, including flagellates with a pointed posterior end and swollen central region (Fig. 7f–h), which progressively turn into forms with a blunt posterior extremity until whole differentiation into bell-shaped flagellates with long free flagella (Fig. 7f, g, k), which correspond to the apparently ‛roundedʼ metacyclic trypomastigotes observed by light microscopy (Fig. 7c, d).

**Fig. 8:**
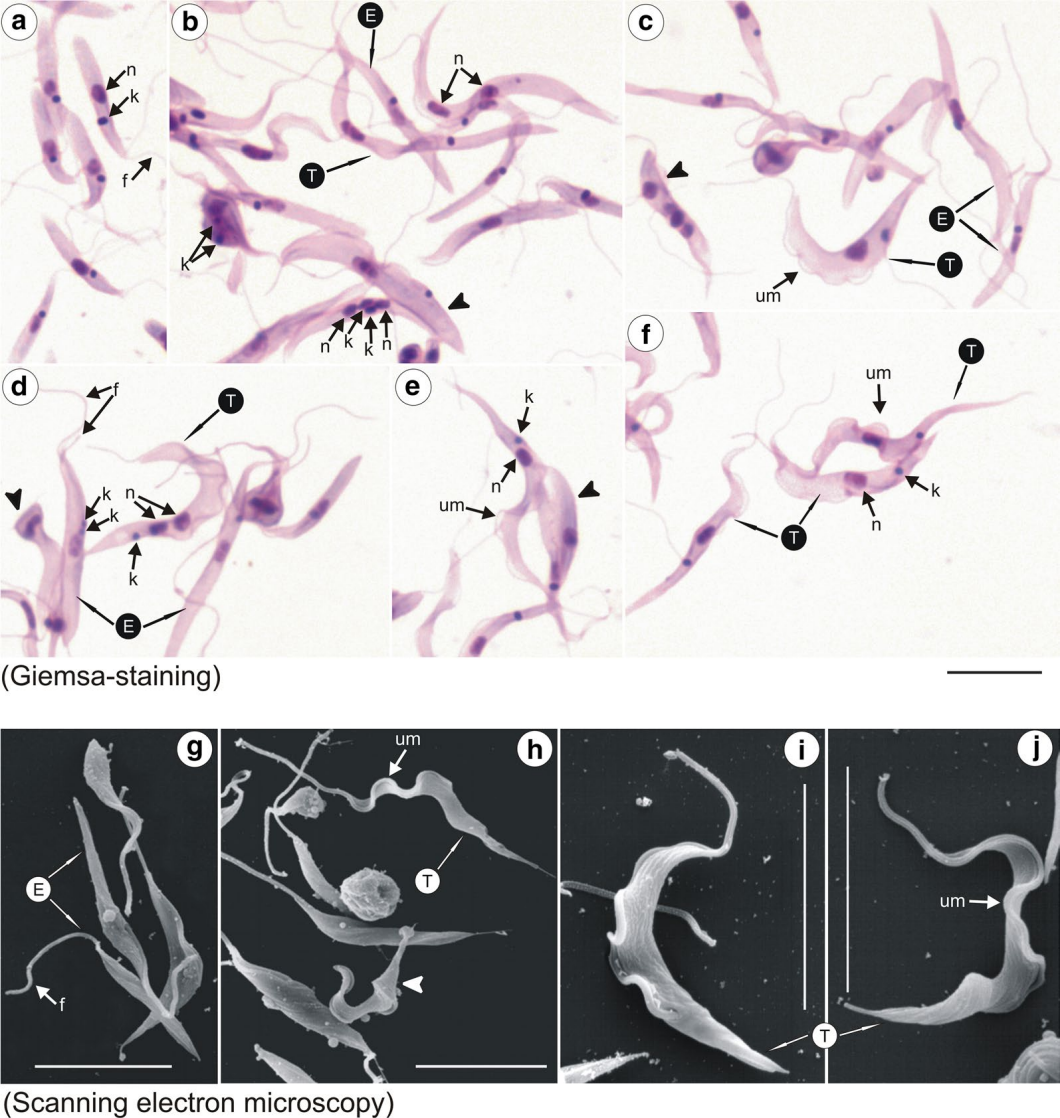
Light (Giemsa-staining) and scanning electron microscopy (SEM) of *Trypanosoma trinaperronei* n. sp. cultured with monolayers of mammalian (LLCMK_2_) cells at 37 °C. Giemsa-stained (**a**–**f**) and SEM (**g**–**j**) showing epimastigotes with one day of culture (**a**, **g**), and developmental forms (5 days): large and wide epimastigotes and trypomastigotes **(b-d)**; flagellates with two nuclei and one kinetoplast (**b, d**); transition forms (arrow heads) between epimastigotes and trypomastigotes (**b, d, e**); and slender and pointed trypomastigotes with a well-developed undulating membrane **(d**–**f, h**–**j).***Abbreviations*: nucleus, n; kinetoplast, k; flagellum, f; undulating membrane, um; epimastigote, E; trypomastigote, T; trypomastigote, T. *Scale-bars*: **a**–**j**,10 µm

Log-phase epimastigotes from Hi5-cultures were seeded into monolayers of mammalian LLC-MK2 cells, incubated at 37 °C with 5% CO_2_, and after one to 5 days, cultures of Giemsa-stained flagellates were examined by light microscopy (Fig. 8a–f) and SEM (Fig. 8g–j). In the supernatant of these cultures, slender epimastigotes gradually became wider (Fig. 8a, g; one day culture) and gave origin to large and wide transition forms (indicated by arrowheads in Fig. 8e, h) between epimastigotes and trypomastigotes initially exhibiting wide bodies (Fig. 8b, c, h), and then becoming long and slender showing well-developed undulating membranes and pointed posterior ends (Fig. 8e, f, i, j). Both large epimastigotes and trypomastigotes are multiplicative forms (Fig. 8b, d). Long and slender forms with sharpened posterior ends and prominent undulating membranes (Fig. 8e, f, i, j) resemble those present in early haemocultures (Fig. 2) as well as blood trypomastigotes of *T. theileri* of cattle and *T. theileri* -like of water buffalo [6, 21, 23, 53, 63], and *T. cervi* and *T. cervi*-like [2, 6, 10, 38, 64]. Intracellular rounded flagellates resembling ‘amastigotes’ [65] could be observed inside mammalian cells (data not shown), but unquestionable demonstration of their intracellular development and differentiation requires further studies.

Taken together, cultures of *T. trinaperronei* n. sp. showed large epimastigotes and trypomastigotes typical of *Megatrypanum* trypanosomes present in both early haemocultures (Fig. 2) and mammalian cell cultures (Fig. 8), similar to previously reported in deer blood [2, 6, 10, 38, 64]. In addition, clumps of small, rounded forms and epimastigotes detected in early co-cultures of *T. trinaperronei* n. sp. with insect cell (Fig. 7) were quite similar to those reported in guts of deer keds infected with *T.* (*Megatrypanum*) spp. [38]. Altogether, these findings allowed for inferences about the morphological differentiation through the life-cycle of *T. trinaperronei* n. sp. in vertebrate hosts and putative vectors according to the herein predicted life-cycle (Fig. 2). Morphological comparison of *T. trinaperronei* n. sp. blood trypomastigotes and epimastigotes from vector guts with corresponding forms of previously reported trypanosomes in deer species did not revealed species-specific features, thus corroborating the high morphological resemblance of all *Megatrypanum* trypanosomes [2, 6, 10, 21, 38, 64].

### Ultrastructural characterization of *Trypanosoma trinaperronei* n. sp

**Fig. 9:**
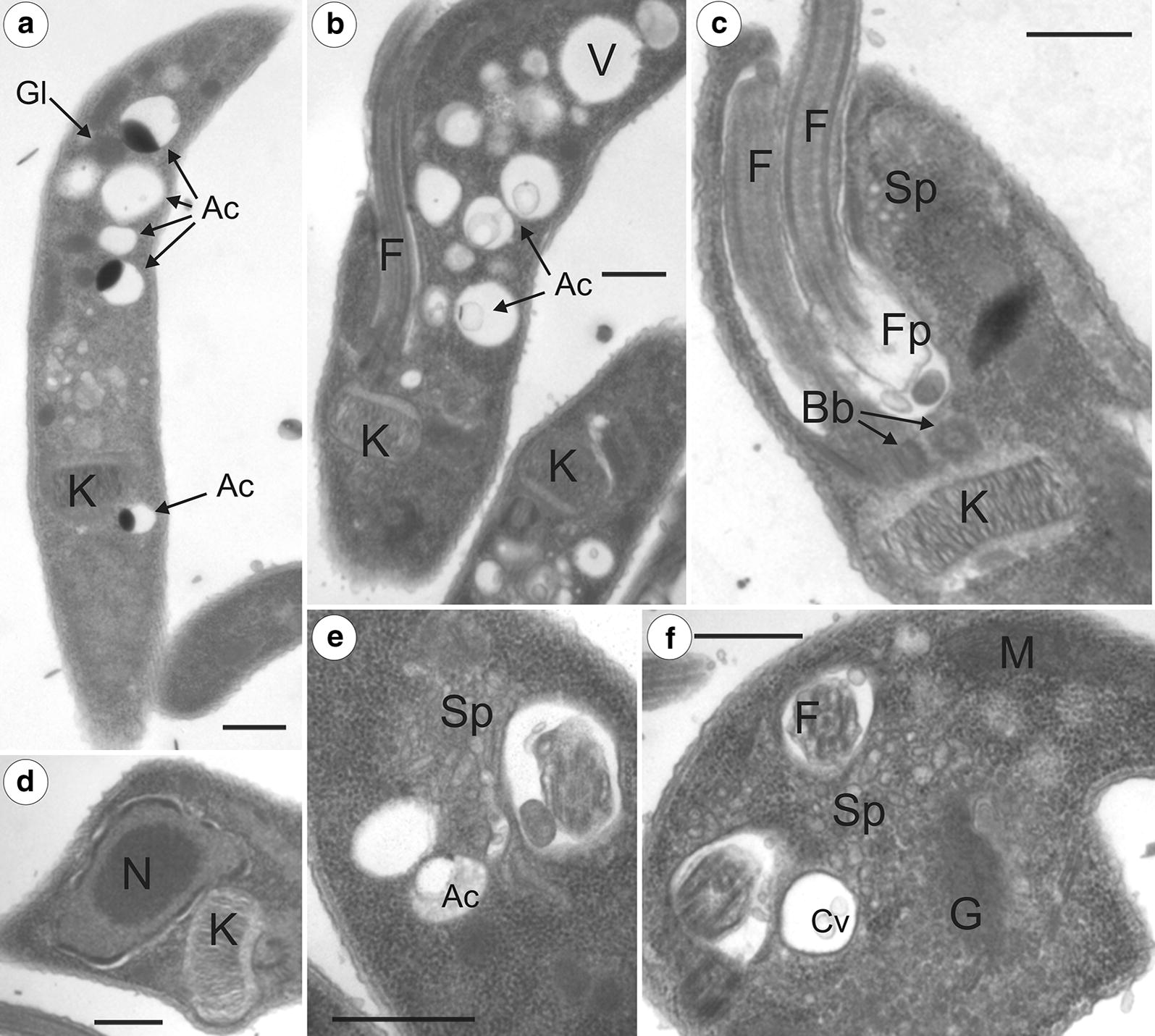
Ultrastructural features of *Trypanosoma trinaperronei* n. sp. revealed by transmission electron microscopy (TEM). Longitudinal section of epimastigote (**a**, **c**) and trypomastigote (**b**) forms showing many acidocalcisomes (**a**, **b**), and kinetoplasts displaying weakly compacted and long DNA fibrils (**a**–**d**). Flagellar pocket region and the spongiome, a network of tubules and large contractile vacuole (**c**, **e**, **f**). Longitudinal section of a dividing epimastigote exhibiting two flagella, two basal bodies, and a single kinetoplast (**c**). *Abbreviations*: nucleus, N; kinetoplast, K; flagellum, F; Golgi, G; glycosome, GI; contractile vacuole, Cv; basal body, Bb; acidocalcisomes, Ac; Mitochondria, M; spongiome, Sp. *Scale-bars*: **a**–**f**, 0.5 µm

Transmission electron microscopy (TEM) of cultured *T. trinaperronei* n. sp. revealed mitochondrion, Golgi, glycosomes, acidocalcisomes, flagellum, and overall ultrastructural organization typical of trypanosomatids. A set of features can be considered common of *Megatrypanum* trypanosomes: an abundance of acidocalcisomes (Fig. 9a, b) distributed throughout the cell body; a kinetoplast exhibiting lengthy and weakly compacted DNA fibrils (Fig. 9a–d); a noticeable spongiome comprising a network of tubules and contractile vacuoles near the flagellar pocket (Fig. 9c, e, f); and the absence of cytostome. To our knowledge, this is the first time that a deer trypanosome is characterized by TEM, and the ultra-structural arrangement was similar to that reported previously for *T. theileri* [23, 65].

### Description of the new species

**Family Trypanosomatidae Doflein, 1951**

**Genus*Trypanosoma*Gruby, 1843**

***Trypanosoma*(*Megatrypanum*)*trinaperronei*Teixeira, Camargo & García n. sp.**

***Type-host***: *Odocoileus virginianus* Zimmermann (Ruminantia, Cervidae), white-tailed deer.

***Type-material***: Hapantotype: the culture of the isolate TCC2268. Paratypes: blood samples of WTDs and gut samples of deer keds preserved in ethanol infected with *Trypanosoma* (*M.*) *trinaperronei* n. sp. Culture of *T. trinaperronei* n. sp. (TCC2268) is cryopreserved at the Trypanosomatid Culture Collection (TCC-USP) of the University of São Paulo, located at the Department of Parasitology, ICB, USP, São Paulo, Brazil, and registered in the World Data Centre for Microorganisms (WDCM611) of the Word Federation for Culture Collection (WFCC, 1981-10-14). TCC/USP also includes Giemsa-stained smears of cultures in glass slides, and blood samples of WTD at BSC (blood sample collection) and gut samples of deer keds at ISC (Insect Sample Collection).

***Type-locality***: State of Anzoátegui (10°07′08.95″N, 64°38′23.80″W), Venezuela.

***Other locality***: Texas, USA.

***Invertebrate host (putative vector)***: *Lipoptena mazamae* Rondani, 1878 (Diptera: Hippoboscidae), deer ked.

***Site in vertebrate host***: Blood.

***Site in invertebrate host***: Digestive tract.

***Representative DNA sequences***: *Trypanosoma* (*M.*) *trinaperronei* n. sp. (TCC2268) DNA sequences deposited in the GenBank database as follows: *SSU* rRNA (MN752212); V7V8*SSU* rRNA (MN752143); *gGAPDH* (MN756794); ITS1 rDNA (MN752208, MN752209); and *CATL* (MN747149-MN747155). DNA sequences of *Trypanosoma* sp. PJH-2013a (isolate WTD A3) from the blood of WTD from Texas, USA herein designed as a genotype of *T. trinaperronei* n. sp.: *SSU* rRNA and ITS rDNA (JX178172-JX178173).

***ZooBank registration***: To comply with the regulations set out in Article 8.5 of the amended 2012 version of the *International Code of Zoological Nomenclature* (ICZN) [66], details of the new species have been submitted to ZooBank. The Life Science Identifier (LSID) of the article is urn:lsid:zoobank.org:pub:0F4D44A3-E37D-4687-9DC6-9797E503E3C3.

***Etymology***: The name “trinaperronei” was given as a tribute to Dr Trina Mercedes Perrone Carmona, a Venezuelan biologist who contributed to the knowledge of animal trypanosomiasis and the progress of the veterinary sciences in Venezuela, and who died unexpectedly in 2008.

**Description**

Log-phase forms of *T. trinaperronei* n. sp. co-cultured with Hi-5 insect cells and examined by light microscopy and SEM were epimastigotes (*n* = 30) with body size averaging 21.61 ± 9.11 µm in length (range 13.42–47.02 µm) and 2.35 ± 1.03 µm in width (range 1.29–5.3 µm), lacking conspicuous undulating membranes and exhibiting a long (mean: 20.15 ± 6.35 µm; range: 11.90–30.3 µm) free flagellum in log-phase cultures (Fig. 7a–d); ‛roundedʼ forms with a posterior kinetoplast and long flagellum were observed in stationary-phase cultures (Fig. 7b–d). *Trypanosoma trinaperronei* n. sp. co-cultured with mammalian cells showed large and wide epimastigotes and trypomastigotes, and long and slender trypomastigotes with pointed posterior end, all forms exhibiting noticeable undulating membrane. Ultrastructural features (TEM) of *T. trinaperronei* n. sp. are typical of all *Megatrypanum* trypanosomes (Fig. 9).

**Remarks**

*Trypanosoma trinaperronei* n. sp. was detected in WTD from Venezuela (isolate WTD TCC2268) and USA (isolate WTD A3). *SSU* rRNA and ITS rDNA sequences of these two isolates diverged by 0.3 and 0.6%, respectively. The new species *T. trinaperronei* n. sp. and its closest relative *Trypanosoma* sp. D30 of fallow deer from Germany (both positioned in the TthII lineage of the subgenus *Megatrypanum*) diverged by 0.5%, 2.2% and 17% on *SSU* rRNA, *gGAPDH* and ITS rDNA sequences, respectively. Recent phylogenies detected DNA of a trypanosome of the TthI lineage in WTD and elk from USA [12]. *Trypanosoma mazamarum*, described in the blood of brocket deer (*Mazama* sp.) restricted to South America [19] exhibited large blood trypomastigotes typical of all species of the subgenus *Megatrypanum*, but neither cultures nor DNA sequences of this trypanosome are available for comparison with the molecularly characterized trypanosomes of this subgenus, which are virtually morphologically indistinguishable trypanosomes from bovids and cervids.

## Conclusions

In the present study, we combined molecular, morphological, behaviour in cultures, biological and phylogeographical data to describe *T. trinaperronei* n. sp. detected in WTD blood and deer keds. Phylogeographical histories of cervids and respective trypanosomes from this and previous studies support historical dispersion of cervids and co-migrating *Megatrypanum* trypanosomes. Altogether, deer-trypanosome-vector associations and phylogeography of both trypanosomes and deer support a plausible evolutionary scenario where WTD infected with the ancestor of *T. trinaperronei* n. sp., likely infested by its tightly linked ectoparasite *L. mazamae*, were introduced from North America into South America through the Panama Isthmus. This scenario is strongly reinforced by the discovery of recently diversified geographical variants of the Pan-American *T. trinaperronei* n. sp. in the USA and Venezuela, compatible with the introduction of *T. trinaperronei* n. sp. through the recent invasion of South America by WTD from North America at the Pliocene-Pleistocene boundary. Underestimated genetic repertoire and entangled relationships of morphologically indistinguishable trypanosomes from cervids and bovids highlight the need for more comprehensive surveys to assess species richness, and host-parasite-vector associations of *Megatrypanum* trypanosomes.

## Supplementary information

**Additional file 1: Table S1.** Isolates of trypanosomes of the subgenus *Megatrypanum* employed for network inferences using cathepsin L sequences (*CATL*).

**Additional file 2: Table S2.** Isolates of trypanosomes of the subgenus *Megatrypanum* employed for phylogenetic inferences using *SSU* rRNA sequences.

**Additional file 3: Table S3.** Isolates of trypanosomes of the subgenus *Megatrypanum* employed for phylogenetic inferences using *gGAPDH* sequences.

**Additional file 4: Table S4.** Isolates of trypanosomes of the subgenus *Megatrypanum* employed for Network inferences using ITS1 rDNA sequences.

## Data Availability

Cultured flagellates cryopreserved and in glass slides, and DNA samples of *Trypanosoma trinaperronei* n. sp. are deposited at the Trypanosomatid Culture Collection (TCC-USP) of the Department of Parasitology, ICB, USP, São Paulo, Brazil, under the accession number TCC2268. The newly generated DNA sequences were deposited in the GenBank database under the accession numbers MN752212 (*SSU* rRNA), MN752143 (V7V8 *SSU* rRNA), MN756794 (*gGAPDH*), MN752208, MN752209 (ITS1 rDNA) and MN747149-MN747155 (*CATL* gene).

## Abbreviations

TCC: Trypanosomatid Culture Collection of the Department of Parasitology, ICB, University of São Paulo, SP, Brazil
*SSU* rRNA: small subunit of ribosomal RNA
*gGAPDH*: glycosomal glyceraldehyde-3-phosphate dehydrogenase
*CATL*: cathepsin L
ITS rDNA: internal transcribed spacer of rDNA
PCR: polymerase chain reaction
Hi-5: insect (*Trichoplusia* sp.) cell
WTD: white-tailed deer
mya: million years ago
