# Supplementary material for: Pan-American Trypanosoma (Megatrypanum) trinaperronei n. sp. in the white-tailed deer Odocoileus virginianus Zimmermann and its deer ked Lipoptena mazamae Rondani, 1878: morphological, developmental and phylogeographical characterisation
Source: Parasit Vectors. 2020 Jun 12;13:308. doi: 10.1186/s13071-020-04169-0 (PMC7291487; doi:10.1186/s13071-020-04169-0)
Supplement: Supplementary file 1 — Additional file 1: Table S1. Isolates of trypanosomes of the subgenus Megatrypanum employed for network inferences using cathepsin L sequences (CATL). [file 13071_2020_4169_MOESM1_ESM.doc]

**Additional Table S1**

Isolates of trypanosomes of the subgenus *Megatrypanum* employed for Network inferences using cathepsin L sequences (*CATL*).

| **Phylogenetic lineage/genotype** | **GenBank**  **Accession number** | **Isolate identification** | **Host origin** | **Country** |
| --- | --- | --- | --- | --- |
| TthI A | GU299375 | Tthb2c3 | buffalo | Brazil |
|  | GU299388 | Tthb3c4 | buffalo | Brazil |
|  | GU299378 | Tthb4c1 | buffalo | Brazil |
|  | GU299379 | Tthb4c2 | buffalo | Brazil |
|  | GU299380 | Tthb4c3 | buffalo | Brazil |
|  | GU299383 | Tthb9c2 | buffalo | Brazil |
|  | GU299381 | Tthb10c2 | buffalo | Brazil |
|  | HQ664732 | Tthb12c2 | buffalo | Brazil |
|  | HQ664733 | Tthb13c2 | buffalo | Brazil |
|  | HQ664734 | Tthb17c3 | buffalo | Brazil |
|  | HQ664735 | Tthb19c3 | buffalo | Brazil |
|  | HQ664736 | TthbV9c2 | buffalo | Venezuela |
|  | HQ664737 | TthbV13c3 | buffalo | Venezuela |
|  | HQ664738 | TthbV15c1 | buffalo | Venezuela |
|  | AB930161 | MN223 | buffalo | Sri Lanka |
|  | AB930166 | MN132 | buffalo | Sri Lanka |
| TthI B | GU299400 | Tthc1c2 | cattle | Brazil |
|  | GU299397 | Tthc2c1 | cattle | Brazil |
|  | GU299398 | Tthc2c4 | cattle | Brazil |
|  | HQ664739 | Tthc3c1 | cattle | Brazil |
|  | GU299391 | TthATCCc1 | cattle | USA |
|  | AB742560 | Tth-ca-no.42 | cattle | Vietnam |
|  | AB742561 | Tth-ca-no.43 | cattle | Vietnam |
|  | AB930145 | T5 | cattle | Sri Lanka |
|  | AB930150 | P69 | cattle | Sri Lanka |
|  | JX860299 | 41-42 | cattle | Philippines |
|  | JX860300 | 43-44 | cattle | Philippines |
|  | LC125448 | CA95 | cattle | Vietnam |
|  | LC125447 | CA84 | cattle | Vietnam |
| TthI C | GU299401 | Tthc9c1 | cattle | Brazil |
|  | GU299404 | Tthc10c3 | cattle | Brazil |
|  | GU299405 | Tthc8c1 | cattle | Brazil |
|  | GU299406 | Tthc8c5 | cattle | Brazil |
| TthI D | AB930146 | T39 | cattle | Sri Lanka |
|  | JX860298 | 37-38 | cattle | Philippines |
| TthI E | LC125451 | CA136 | cattle | Vietnam |
|  | AB930148 | P12 | cattle | Sri Lanka |
| TthI F | AB742562 | Tth-ca-no.90 | cattle | Vietnam |
|  | AB742559 | Tth-ca-no.28 | cattle | Vietnam |
|  | AB930155 | AK1 | cattle | Sri Lanka |
| TthI G | AB930154 | AM20 | cattle | Sri Lanka |
| TthI H | AB930151 | AT9 | cattle | Sri Lanka |
| TthI I (former Thai Ib) | AB930162 | MN39 | buffalo | Sri Lanka |
|  | HQ543073 | KS34c6 | cattle | Thailand |
|  | HQ543074 | KS34c3 | cattle | Thailand |
| TthI J | AB930163 | MN60 | buffalo | Sri Lanka |
|  | AB930164 | MN73 | buffalo | Sri Lanka |
| TthI K (former Thai Ia) | HQ543061 | KA24c2 | cattle | Thailand |
|  | HQ543068 | KA24c6 | cattle | Thailand |
|  | HQ543063 | KS46c10 | cattle | Thailand |
|  | HQ543069 | A4c5 | cattle | Thailand |
|  | HQ543071 | KS46c2 | cattle | Thailand |
|  | HQ543072 | KA24c4 | cattle | Thailand |
|  | AB930157 | J8 | cattle | Sri Lanka |
|  | AB930149 | P38 | cattle | Sri Lanka |
|  | LC125450 | CA119c2 | cattle | Vietnam |
| TthI L | LC125446 | CA84 | cattle | Vietnam |
| TthII A | GU299354 | Tthc30c2 | cattle | Brazil |
|  | GU299355 | Tthc30c5 | cattle | Brazil |
|  | GU299351 | Tthc32c2 | cattle | Brazil |
|  | GU299352 | Tthc32c3 | cattle | Brazil |
|  | GU299347 | Tthc37c4 | cattle | Brazil |
|  | GU299348 | Tthc37c5 | cattle | Brazil |
| TthII B | GU299395 | Tthc16c2 | cattle | Brazil |
|  | GU299371 | Tthc5c3 | cattle | Brazil |
|  | GU299368 | Tthc14c2 | cattle | Brazil |
|  | GU299373 | Tthc19c3 | cattle | Brazil |
|  | GU299359 | Tthc28c1 | cattle | Brazil |
|  | HQ664740 | Tthc39c3 | cattle | Brazil |
|  | HQ664741 | Tthc40c2 | cattle | Brazil |
|  | HQ664742 | Tthc41c1 | cattle | Brazil |
|  | HQ664743 | TthcV2c1 | cattle | Venezuela |
|  | HQ664744 | TthcV4c3 | cattle | Venezuela |
|  | AB930159 | J34 | cattle | Sri Lanka |
|  | AB930158 | J29 | cattle | Sri Lanka |
|  | AB742558 | Tth-ca-no.10 | cattle | Vietnam |
|  | LC125453 | BU15 | buffalo | Vietnam |
|  | LC125455 | BU50 | buffalo | Vietnam |
|  | LC125452 | CA141 | cattle | Vietnam |
| TthII C | GU299415 | TspD30c1 | fallow deer | Germany |
|  | GU299416 | TspD30c2 | fallow deer | Germany |
|  | GU299417 | TspD30c4 | fallow deer | Germany |
| **TthII D** | **MN756796 *** | **TmHR1c1** | **sheep ked** | **Croatia** |
|  | **MN756797 *** | **TmHR1c2** | **sheep ked** | **Croatia** |
| TthII E | HQ664745 | SitaBip1c1 | sitatunga | Cameroon |
|  | HQ664746 | SitaBip1c2 | sitatunga | Cameroon |
|  | HQ664747 | SitaBip1c4 | sitatunga | Cameroon |
| TthII F | HQ664748 | CepCamp4c1 | duiker | Cameroon |
|  | HQ664749 | CepCamp5c1 | duiker | Cameroon |
|  | HQ664750 | CepCamp5c2 | duiker | Cameroon |
| Tth II G | HQ664751 | CepCamp5c4 | duiker | Cameroon |
| **TthII H** | **MN747149 *** | **TCC2268c1** | **WTD** | **Venezuela** |
|  | **MN747150 *** | **TCC2268c2** | **WTD** | **Venezuela** |
|  | **MN747151 *** | **TCC2268c3** | **WTD** | **Venezuela** |
|  | **MN747152 *** | **TCC2268c7** | **WTD** | **Venezuela** |
|  | **MN747153 *** | **TCC2268c8** | **WTD** | **Venezuela** |
|  | **MN747154 *** | **cLM1** | **deer ked** | **Venezuela** |
|  | **MN747155 *** | **cLM2** | **deer ked** | **Venezuela** |
| TthII Thai IIa | HQ543060 | MK81c1 | cattle | Thailand |
|  | HQ543057 | MK81c3 | cattle | Thailand |

*** Sequences determined in this study**
