# Supplementary material for: Pan-American Trypanosoma (Megatrypanum) trinaperronei n. sp. in the white-tailed deer Odocoileus virginianus Zimmermann and its deer ked Lipoptena mazamae Rondani, 1878: morphological, developmental and phylogeographical characterisation
Source: Parasit Vectors. 2020 Jun 12;13:308. doi: 10.1186/s13071-020-04169-0 (PMC7291487; doi:10.1186/s13071-020-04169-0)
Supplement: Supplementary file 2 — Additional file 2: Table S2. Isolates of trypanosomes of the subgenus Megatrypanum employed for phylogenetic inferences using SSU rRNA sequences. [file 13071_2020_4169_MOESM2_ESM.doc]

**Additional Table S2**

Isolates of trypanosomes of the subgenus *Megatrypanum* employed for phylogenetic inferences using *SSU* rRNA sequences.

| **Phylogenetic lineage** | **GenBank Accession number** | **Isolate identification** | **Host origin** | **Country** |
| --- | --- | --- | --- | --- |
| **Tth I** | HQ664904 | TthbV15 | buffalo | Venezuela |
|  | HQ664903 | TthbV13 | buffalo | Venezuela |
|  | HQ664902 | TthbV9 | buffalo | Venezuela |
|  | HQ664901 | Tthb22 | buffalo | Brazil |
|  | HQ664900 | Tthb20 | buffalo | Brazil |
|  | HQ664899 | Tthb19 | buffalo | Brazil |
|  | HQ664898 | Tthb17 | buffalo | Brazil |
|  | HQ664897 | Tthb16 | buffalo | Brazil |
|  | HQ664896 | Tthb14 | buffalo | Brazil |
|  | AY773678 | Tthb13 | buffalo | Brazil |
|  | AY773677 | Tthb12 | buffalo | Brazil |
|  | HQ664895 | Tthb10 | buffalo | Brazil |
|  | AY773676 | Tthb6 | buffalo | Brazil |
|  | AY773675 | Tthb4 | buffalo | Brazil |
|  | AY773674 | Tthb3 | buffalo | Brazil |
|  | HQ664490 | Tthc1 | cattle | Brazil |
|  | AY773679 | Tthc2 | cattle | Brazil |
|  | AY773681 | Tthc3 | cattle | Brazil |
|  | AY773682 | Tthc8 | cattle | Brazil |
|  | AY773683 | Tthc9 | cattle | Brazil |
|  | AY773684 | Tthc10 | cattle | Brazil |
|  | AY773686 | Tthc15 | cattle | Brazil |
|  | AY773687 | Tthc16 | cattle | Brazil |
|  | JX853185 | Cow2095.c4 | cattle | USA |
|  | JX178185 | Cow139.c11 | cattle | USA |
|  | JX178166 | Cow2.c10 | cattle | USA |
|  | JX178162 | Cow2095.c8 | cattle | USA |
|  | AB007814 | KM | cattle | Japan |
|  | KU587639 | CBT113 | cattle | Brazil |
|  | KU587648 | CBT137 | cattle | Brazil |
|  | JX178188 | Cow104.c2 | cattle | USA |
|  | JX178184 | Cow139.c10 | cattle | USA |
|  | JX178168 | Cow3535.c6 | cattle | USA |
|  | JX178187 | Cow104.c1 | cattle | USA |
|  | JX178183 | Cow139.c9 | cattle | USA |
|  | JX853183 | WTD A1.c7 | WTD | USA |
|  | JX178192 | WTD A1.c1 | WTD | USA |
|  | AB569248 | TSD1 | sika deer | Japan |
|  | KF765799 | Bb813 | wisent | Poland |
|  | KR024688 | G24 | tse-tse | Central African Republic |
|  | MK088729 | TrypTab5 | tabanid | Poland |
|  | MK088731 | TrypTab65 | tabanid | Poland |
|  | JX178197 | WTD 148.c4 | WTD | USA |
|  | JX178199 | ELK 328.c21 | Elk | USA |
|  | JX178196 | WTD A5.c1 | WTD | USA |
|  | JX853182 | WTD A1.c6 | WTD | USA |
|  | JX178177 | ELK 142.c9 | Elk | USA |
|  | JX178179 | ELK 416.c8 | Elk | USA |
|  | JX178178 | ELK 142.c10 | Elk | USA |
|  | JX853184 | ELK 421.c2 | Elk | USA |
|  | JX178194 | WTD A21.c4 | WTD | USA |
|  | JX178171 | WTD NL15.c9 | WTD | USA |
|  | JX178169 | WTD NL15.c1 | WTD | USA |
|  | MK156794 | NovSl2 | tabanid | Russia |
|  | MK156793 | KrSl7 | tabanid | Russia |
|  | MK156792 | KrSl4 | tabanid | Russia |
|  | JX178175 | WTD A21.c5 | WTD | USA |
|  | JX178176 | WTD A21.c6 | WTD | USA |
|  | JX178195 | WTD A21.c6 | WTD | USA |
|  | JX178180 | ELK 328.c3 | WTD | USA |
|  | JX178174 | WTD A21.c4 | WTD | USA |
|  | KF805968 | Cel14St | red deer | Poland |
|  | KJ195884 | DdP287 | fallow deer | Poland |
| **Tth II** | GQ176159 | Tthc37 | cattle | Brazil |
|  | GQ176158 | Tthc32 | cattle | Brazil |
|  | GQ176157 | Tthc30 | cattle | Brazil |
|  | KU587643 | CBT120 | cattle | Brazil |
|  | KU587646 | CBT123 | cattle | Brazil |
|  | AY773689 | Tthc5 | cattle | Brazil |
|  | AY773690 | Tthc12 | cattle | Brazil |
|  | AY773691 | Tthc13 | cattle | Brazil |
|  | AY773692 | Tthc14 | cattle | Brazil |
|  | AY773693 | Tthc17 | cattle | Brazil |
|  | AY773694 | Tthc18 | cattle | Brazil |
|  | AY773695 | Tthc19 | cattle | Brazil |
|  | AY773696 | Tthc20 | cattle | Brazil |
|  | AY77369X | Tthc21 | cattle | Brazil |
|  | GQ176152 | Tthc24.c12 | cattle | Brazil |
|  | GQ176154 | Tthc25 | cattle | Brazil |
|  | GQ176153 | Tthc26 | cattle | Brazil |
|  | GQ176160 | Tthc38 | cattle | Brazil |
|  | HQ664906 | Tthc39 | cattle | Brazil |
|  | HQ664907 | Tthc40 | cattle | Brazil |
|  | HQ664908 | Tthc41 | cattle | Brazil |
|  | HQ664910 | TthcV4 | cattle | Venezuela |
|  | HQ664911 | TthcV5 | cattle | Venezuela |
|  | AY971802 | Tab1.2cl2 | tabanid | Brazil |
|  | AY971803 | Tab12.1cl2 | tabanid | Brazil |
|  | KU587631 | CBT138 | cattle | Brazil |
|  | KY009590 | SMA693 | cattle | Argentina |
|  | AB569250 | Esashi12 | cattle | Japan |
|  | AB569249 | Esashi9 | cattle | Japan |
|  | JX178162 | Cow2073.c9 | cattle | Brazil |
|  | HQ664909 | TthcV2 | cattle | Venezuela |
|  | GQ176156 | Tthc29 | cattle | Brazil |
|  | AJ009164 | K127 | cattle | United Kingdom |
|  | LC385952 | Obihiro | cattle | Japan |
|  | KU587641 | CBT115 | cattle | Brazil |
|  | KY009595 | SMA288 | cattle | Argentina |
|  | KY009592 | SMC3179 | cattle | Argentina |
|  | HQ664842 | SitaBip1 | sitatunga | Cameroon |
|  | HQ664832 | CepCamp4 | duiker | Cameroon |
|  | HQ664837 | CapCamp5 | duiker | Cameroon |
|  | FN666409 | St_Kilda | sheep ked | United Kingdom |
|  | HQ664912 | TmHR1 | deer ked | Croatia |
|  | MK088728 | TrypHpl10Sach | tabanid | Poland |
|  | KY681802 | 2016/SF5 | sand fly | Italy |
|  | MK156791 | KrSl1 | tabanid | Russia |
|  | KJ397590 | Cel34 | red deer | Poland |
|  | KJ195885 | DdP18 | fallow deer | Poland |
|  | KJ195879 | Cn1 | sika deer | Poland |
|  | JN798594 | TC2 | red deer | Croatia |
|  | AJ009165 | Tsp.D30 | fallow deer | Germany |
|  | MK088730 | TrypHpl13Sach | tabanid | Poland |
|  | **MN752212 *** | **TCC2268** | **WTD** | **Venezuela** |
|  | JX178172 | WTD A3.c9 | WTD | USA |
|  | JX178198 | ELK 328.c20 | WTD | USA |

*** Sequences determined in this study**
