# Supplementary material for: Pan-American Trypanosoma (Megatrypanum) trinaperronei n. sp. in the white-tailed deer Odocoileus virginianus Zimmermann and its deer ked Lipoptena mazamae Rondani, 1878: morphological, developmental and phylogeographical characterisation
Source: Parasit Vectors. 2020 Jun 12;13:308. doi: 10.1186/s13071-020-04169-0 (PMC7291487; doi:10.1186/s13071-020-04169-0)
Supplement: Supplementary file 3 — Additional file 3: Table S3. Isolates of trypanosomes of the subgenus Megatrypanum employed for phylogenetic inferences using gGAPDH sequences. [file 13071_2020_4169_MOESM3_ESM.doc]

**Additional Table S3**

Isolates of trypanosomes of the subgenus *Megatrypanum* employed for phylogenetic inferences using *gGAPDH* sequences.

| **Phylogenetic lineage/genotype** | **GenBank Accession number** | **Isolate identification** | **Host origin** | **Country** |
| --- | --- | --- | --- | --- |
| TthI A | HQ664791 | TthbV15 | buffalo | Venezuela |
|  | HQ664790 | Tthb22 | buffalo | Brazil |
|  | HQ664788 | Tthb19 | buffalo | Brazil |
|  | HQ664786 | Tthb16 | buffalo | Brazil |
|  | HQ664787 | Tthb17 | buffalo | Brazil |
|  | HQ664789 | Tthb20 | buffalo | Brazil |
|  | HQ664785 | Tthb14 | buffalo | Brazil |
|  | HQ664784 | Tthb10 | buffalo | Brazil |
|  | HQ664792 | Tthc1 | cattle | Brazil |
|  | HQ664793 | Tthc3 | cattle | Brazil |
| TthII A | HQ664801 | Tthc30 | cattle | Brazil |
|  | HQ664802 | Tthc32 | cattle | Brazil |
|  | HQ664803 | Tthc37 | cattle | Brazil |
| TthII B | AJ620282 | K127 | cattle | Japan |
|  | HQ664799 | Tthc29 | cattle | Brazil |
|  | HQ664796 | Tthc41 | cattle | Brazil |
|  | HQ664795 | Tthc40 | cattle | Brazil |
|  | HQ664798 | TthcV5 | cattle | Venezuela |
|  | HQ664797 | TthcV4 | cattle | Venezuela |
|  | HQ664794 | Tthc39 | cattle | Brazil |
| TthII C | HQ664806 | Tsp.D30 | fallow deer | Germany |
| TthII D | HQ664807 | TmHR1 | deer ked | Croatia |
| TthII E | FM164792 | SitaBip1 | sitatunga | Cameroon |
| TthII F | HQ664804 | CepCamp4 | duiker | Cameroon |
| TthII G | HQ664805 | CepCamp5 | duiker | Cameroon |
| **TthII H** | **MN756794 *** | **TCC2268** | **WTD** | **Venezuela** |

*** Sequences determined in this study**
