# Supplementary material for: Pan-American Trypanosoma (Megatrypanum) trinaperronei n. sp. in the white-tailed deer Odocoileus virginianus Zimmermann and its deer ked Lipoptena mazamae Rondani, 1878: morphological, developmental and phylogeographical characterisation
Source: Parasit Vectors. 2020 Jun 12;13:308. doi: 10.1186/s13071-020-04169-0 (PMC7291487; doi:10.1186/s13071-020-04169-0)
Supplement: Supplementary file 4 — Additional file 4: Table S4. Isolates of trypanosomes of the subgenus Megatrypanum employed for Network inferences using ITS1 rDNA sequences. [file 13071_2020_4169_MOESM4_ESM.doc]

**Additional Table S4**

Isolates of trypanosomes of the subgenus *Megatrypanum* employed for Network inferences using ITS1 rDNA sequences.

| **Phylogenetic lineage/genotype** | **GenBank Accession number** | **Isolate identification** | **Host origin** | **Country** |
| --- | --- | --- | --- | --- |
| TthI A | HQ664809 | Tthb14 | buffalo | Brazil |
|  | AY773702 | Tthb12 | buffalo | Brazil |
|  | HQ664808 | Tthb10 | buffalo | Brazil |
|  | HQ664815 | TthbV13 | buffalo | Venezuela |
|  | AY773701 | Tthb4 | buffalo | Brazil |
|  | AY773703 | Tthb13 | buffalo | Brazil |
|  | MF142318 | 514.23 | buffalo | Colombia |
|  | MF142317 | 0.1 | buffalo | Colombia |
|  | MF142315 | 514.1 | buffalo | Colombia |
|  | MF142314 | 75.1 | buffalo | Colombia |
|  | MF142316 | 13S | buffalo | Colombia |
|  | MF142319 | 517.1 | buffalo | Colombia |
|  | MF142313 | 9S | buffalo | Colombia |
| TthI B | AY773706 | Tthc10 | cattle | Brazil |
|  | AY773705 | Tthc9 | cattle | Brazil |
|  | HQ664855 | TthHR1c4 | cattle | Croatia |
|  | HQ664853 | TthHR1c1 | cattle | Croatia |
|  | HQ664854 | TthHR1c2 | cattle | Croatia |
|  | KY412803 | BovAT-SZ-2013 | cattle | Austria |
|  | MK163554 | IZSSI_PA46413 | cattle | Italy |
|  | JX178165 | Cow2c7 | cattle | USA |
|  | JX178183 | Cow139c9 | cattle | USA |
|  | JX178186 | Cow133c1 | cattle | USA |
|  | AB007814 | km | cattle | Japan |
|  | HQ664817 | Tthc1 | cattle | Brazil |
|  | AY773707 | Tthc2 | cattle | Brazil |
|  | AY773698 | Tthc3 | cattle | Brazil |
|  | JX178184 | Cow139c10 | cattle | USA |
|  | JX178166 | Cow2c10 | cattle | USA |
|  | JX178167 | Cow3535c3 | cattle | USA |
|  | AY773708 | Tthc16 | cattle | Brazil |
|  | JX178185 | Cow139c11 | cattle | USA |
|  | JX178168 | Cow3535c6 | cattle | USA |
|  | JX178164 | Cow2c4 | cattle | USA |
|  | KR092365 | JV-2015_TA12_22 | chimpanzee | Cote d'Ivoire |
| TthI C | JX178189 | Cow104c3 | cattle | USA |
|  | JX178187 | Cow104c1 | cattle | USA |
|  | JX178188 | Cow104c2 | cattle | USA |
|  | MG255207 | 237-11-00015-1-38-10 | cattle | Cameroon |
|  | JX178162 | Cow2095c8 | cattle | USA |
|  | JX178163 | Cow2095c9 | cattle | USA |
|  | JX853185 | Cow2095c4 | cattle | USA |
|  | MG255206 | 237-11-00004-1-38-10 | cattle | Cameroon |
| TthI D | JX178170 | WTDNL15c6 | WTD | USA |
|  | JX178171 | WTDNL15c9 | WTD | USA |
|  | JX178169 | WTDNL15c1 | WTD | USA |
|  | JX853182 | WTDA1c6 | WTD | USA |
|  | JX853183 | WTDA1c7 | WTD | USA |
|  | JX178175 | WTDA21c5 | WTD | USA |
|  | JX178174 | WTDA21c4 | WTD | USA |
|  | JX178180 | Elk328c3 | Elk | USA |
|  | JX178179 | Elk416c8 | Elk | USA |
|  | JX178176 | WTDA21c6 | WTD | USA |
| TthI E | JX853184 | Elk421c2 | Elk | USA |
|  | JX178178 | Elk142c10 | Elk | USA |
|  | JX178177 | Elk142c9 | Elk | USA |
| TthI F | AB569248 | TSD1 | sika deer | Japan |
| TthII A | HQ664819 | Tthc30c4 | cattle | Brazil |
|  | GQ176146 | Tthc30c3 | cattle | Brazil |
|  | HQ664820 | Tthc32c4 | cattle | Brazil |
|  | HQ664821 | Tthc32c5 | cattle | Brazil |
|  | GQ176147 | Tthc32c6 | cattle | Brazil |
|  | GQ176148 | Tthc37c2 | cattle | Brazil |
|  | HQ664822 | Tthc37c5 | cattle | Brazil |
| TthII B | MG283143 | 237-11-00361-1-38-10 | cattle | Cameroon |
|  | MF142301 | 9Sc1 | cattle | Colombia |
|  | MF142303 | 1440c1 | cattle | Colombia |
|  | MF142304 | 1289c1 | cattle | Colombia |
|  | MF142305 | 11Sc1 | cattle | Colombia |
|  | MG255208 | 237-11-00165-1-38-10 | cattle | Cameroon |
|  | MF142307 | 1290c1 | cattle | Colombia |
|  | MF142308 | 87c1 | tabanid | Colombia |
|  | MF142323 | 5.1c1 | tabanid | Colombia |
|  | MF142309 | 5c1 | tabanid | Colombia |
|  | LC385951 | Obihiro | cattle | Japan |
|  | HQ664847 | Treu124c2 | cattle | Scotland |
|  | HQ664848 | Treu124c3 | cattle | Scotland |
|  | HQ664849 | Treu124c5 | cattle | Scotland |
|  | AY773710 | Tthc19 | cattle | Brazil |
|  | AY773709 | Tthc14 | cattle | Brazil |
|  | AB569250 | Esashi12 | cattle | Japan |
|  | AB569249 | Esashi9 | cattle | Japan |
|  | MF142320 | 118c1 | cattle | Colombia |
|  | MF142322 | 867.2c1 | cattle | Colombia |
|  | MF142321 | 58c1 | cattle | Colombia |
|  | MF142306 | 10c1 | cattle | Colombia |
|  | MF142302 | 867.1c1 | cattle | Colombia |
|  | MF142312 | 90c1 | cattle | Colombia |
|  | MF142311 | 89c1 | cattle | Colombia |
|  | MF142310 | 88c1 | cattle | Colombia |
|  | AY773711 | Tthc5 | cattle | Brazil |
|  | AY773713 | Tthc18 | cattle | Brazil |
|  | HQ664826 | Tthc40c3 | cattle | Brazil |
|  | GQ176149 | Tthc38c2 | cattle | Brazil |
|  | HQ664823 | Tthc39c4 | cattle | Brazil |
|  | AY773712 | Tthc12c1 | cattle | Brazil |
|  | HQ664829 | TthcV2c7 | cattle | Venezuela |
| TthII C | AY773714 | TspD30 | fallow deer | Germany |
|  | HQ664845 | TspD30c2 | fallow deer | Germany |
|  | JN798601 | TC2 | red deer | Croatia |
|  | KY672996 | 2016/SF5 | sand fly | Italy |
| TthII D | HQ664850 | TmHR1c1 | sheep ked | Croatia |
|  | HQ664851 | TmHR1c4 | sheep ked | Croatia |
|  | HQ664852 | TmHR1c3 | sheep ked | Croatia |
| TthII E | HQ664844 | SitaBip1c4 | sitatunga | Cameroon |
|  | HQ664843 | SitaBip1c2 | sitatunga | Cameroon |
|  | HQ664886 | SitaBip1c1 | sitatunga | Cameroon |
| TthII F | HQ664832 | CepCamp4c2 | duiker | Cameroon |
|  | HQ664834 | CepCamp4c3 | duiker | Cameroon |
|  | HQ664835 | CepCamp4c4 | duiker | Cameroon |
|  | HQ664836 | CepCamp4c5 | duiker | Cameroon |
| TthII G | HQ664839 | CepCamp5c2 | duiker | Cameroon |
|  | HQ664841 | CepCamp5c5 | duiker | Cameroon |
|  | HQ664840 | CepCamp5c4 | duiker | Cameroon |
|  | HQ664837 | CepCamp5c3 | duiker | Cameroon |
| **TthII H** | JX178172 | WTDA3c9.1 | WTD | USA |
|  | JX178173 | WTDA3c9.4 | WTD | USA |
|  | **MN752208 *** | **TCC2268c1** | **WTD** | **Venezuela** |
|  | **MN752209 *** | **TCC2268c2** | **WTD** | **Venezuela** |
| TthII I | JN673397 | ZPU2707 | puku | Zambia |
|  | JN673395 | Z18106 | puku | Zambia |
|  | JN673396 | ZPU2807 | puku | Zambia |

*** Sequences determined in this study**
